# Role of a novel circRNA-CGNL1 in regulating pancreatic cancer progression via NUDT4–HDAC4–RUNX2–GAMT-mediated apoptosis

**DOI:** 10.1186/s12943-023-01923-7

**Published:** 2024-01-31

**Authors:** Hao Yuan, Chuang Chen, Haonan Li, Gexi Qu, Luyao Chen, Yaxing Liu, Yufeng Zhang, Qiang Zhao, Changhong Lian, Aifang Ji, Xuedong Hou, Xinjian Liu, Kuirong Jiang, Yi Zhu, Yuan He

**Affiliations:** 1 Department of General Surgery, Pancreas Centre, the First Affiliated Hospital With Nanjing Medical University, 300 Guangzhou Road, Nanjing, P. R. China; 2 Pancreas Institute, Nanjing Medical University, Nanjing, China; 3 Department of Hepatopancreatobiliary Surgery, The Affiliated Huai’an Hospital of Xuzhou Medical University, Huai’an, China; 4 Changzhi Medical College, Changzhi, China; 5 Heping Hospital, Changzhi Medical College, Changzhi, China; 6 Department of Pathogen Biology, Nanjing Medical University, Nanjing, China

**Keywords:** circCGNL1, NUDT4, HDAC4, Pancreatic cancer

## Abstract

**Background:**

Pancreatic cancer (PC) is an extremely malignant tumor with low survival rate. Effective biomarkers and therapeutic targets for PC are lacking. The roles of circular RNAs (circRNAs) in cancers have been explored in various studies, however more work is needed to understand the functional roles of specific circRNAs. In this study, we explore the specific role and mechanism of circ_0035435 (termed circCGNL1) in PC.

**Methods:**

qRT-PCR analysis was performed to detect circCGNL1 expression, indicating circCGNL1 had low expression in PC cells and tissues. The function of circCGNL1 in PC progression was examined both in vitro and in vivo. circCGNL1-interacting proteins were identified by performing RNA pulldown, co-immunoprecipitation, GST-pulldown, and dual-luciferase reporter assays.

**Results:**

Overexpressing circCGNL1 inhibited PC proliferation via promoting apoptosis. CircCGNL1 interacted with phosphatase nudix hydrolase 4 (NUDT4) to promote histone deacetylase 4 (HDAC4) dephosphorylation and subsequent HDAC4 nuclear translocation. Intranuclear HDAC4 mediated RUNX Family Transcription Factor 2 (RUNX2) deacetylation and thereby accelerating RUNX2 degradation. The transcription factor, RUNX2, inhibited guanidinoacetate N-methyltransferase (GAMT) expression. GAMT was further verified to induce PC cell apoptosis via AMPK–AKT–Bad signaling pathway.

**Conclusions:**

We discovered that circCGNL1 can interact with NUDT4 to enhance NUDT4-dependent HDAC4 dephosphorylation, subsequently activating HDAC4–RUNX2–GAMT-mediated apoptosis to suppress PC cell growth. These findings suggest new therapeutic targets for PC.

**Supplementary Information:**

The online version contains supplementary material available at 10.1186/s12943-023-01923-7.

## Introduction

Pancreatic cancer (PC) is one of the most common malignant tumors of the digestive system and the sixth leading cause of cancer-related deaths in China [1]. Recently, the incidence and mortality of PC have increased, and the 5-year survival rate of PC is only 11% [2–4]. Chemotherapy and surgical resection are used the most to treat PC [5, 6]. However, only 20% of patients with PC are eligible for surgical treatment because effective biomarkers and therapeutic targets for PC are lacking [7, 8]. Therefore, further exploration of the molecular mechanisms and regulatory networks for PC is essential.

Circular RNAs (circRNAs) are a class of noncoding RNAs that commonly exist in the cytoplasm of eukaryotic cells and are mainly derived from exonic regions of protein-coding genes [9]. CircRNAs possess a circular structure without 5'-terminal cap and 3'-terminal poly(A) tail. This structure could avoid the degradation mediated by exonuclease. Hence, it is more stable and conserved than linear RNA [10, 11]. Mounting evidence indicates that interactions between circRNAs and their downstream proteins are crucial for tumor development [12–15]. For example, circ-RanGAP1 regulates VEGFA levels by targeting miR-877-3p, accelerating gastric cancer cell invasion [16]. Circ-HuR can suppress HuR expression and gastric cancer cell growth by inhibiting CNBP transactivation [17]. Many enzymes and receptors are regulated by phosphorylation and dephosphorylation via kinases and phosphatases [18]. Dysfunctional phosphorylation pathways can lead to serious diseases, especially the formation of malignant tumors [19]. NUDT4, as a phosphorylase, was found to interact with circCGNL1 and could mediate the dephosphorylation of HDAC4 in this study. The combination of circCGNL1 with NUDT4 enhances its effect on downstream substrates, which subsequently promotes GAMT expression. GAMT expression is low in PC cells, and might act as a tumor suppressor [20, 21]. As an essential enzyme in the creatine-biosynthetic pathway [22], GAMT can convert the glycine metabolite guanidoacetate to creatine, which mediates AMPK activation [23, 24]. AMPK can induce caspase-family members, leading to cancer cell apoptosis [25, 26], although the corresponding mechanism of GAMT in PC cell apoptosis remains unclear.

The aim of this research was to investigate the biological function and molecular mechanism of circCGNL1 in PC. The interaction between circCGNL1 and the downstream proteins constituted an apoptosis activation signaling pathway, which may help further elucidate the molecular mechanism of PC and provide new biomarkers for PC prognosis.

## Materials and methods

### Patient samples

We obtained paired PC and adjacent tissues from 60 patients in Heping Hospital Affiliated to Changzhi Medical College and 86 patients in the First Affiliated Hospital of Nanjing Medical University. No patient underwent radiotherapy or chemotherapy before surgery, and all patients provided written informed consent, which was approved by the hospital ethics committees (Ethical Number, Heping Hospital Affiliated to Changzhi Medical College: 2023–002, The First Affiliated Hospital of Nanjing Medical University: 2020-SRFA-364). Tissue specimens were collected during operation, cut into two sections within 3 min, and either frozen in liquid nitrogen or fixed in 4% formalin. The overall survival (OS) time was defined as the time between surgery and death. Patients who died within 1 month after surgery were excluded from further analysis.

### Cell culture

Human PC cell lines (MIA PaCa-2, PANC-1, Capan-1, and BxPC-3) and two normal human pancreatic duct epithelial cell lines (HPDE6-C7 and HPNE) were obtained from Cell Bank of Type Culture Collection of the Chinese Academy of Sciences in Shanghai, China. The cells were cultivated in DMEM (MD207-050, Gibco-BRL, USA) containing 10% fetal bovine serum (16000-044, Gibco, USA) and 1% penicillin–streptomycin solution (PR40022, Proteintech, Wuhan, China) at 37 °C with 5% CO_2_. Cell lines underwent routine testing for mycoplasma every 3 months. The genetic identity of the cell lines was confirmed by short tandem repeat profiling.

### RNA isolation, quantitative reverse transcriptase (qRT)-PCR analysis and Sanger sequencing

Total RNA was extracted from cells and PC tissues using TRIzol (15596026, ThermoFisher, USA) and converted to cDNA using PrimeScript Reverse Transcriptase Kit (D7160M, Beyotime, Shanghai, China). According to the manufacturer’s protocol, qPCR was performed using a qRT-PCR kit (QR0100-1KT, Sigma-Aldrich, USA) in a StepOne Plus Real-time PCR System (ThermoFisher). Target gene expressions were detected by the 2^−ΔΔCT^ method, which were standardized with U6. Each qRT-PCR experiment was conducted in triplicate and independently repeated three times. Sanger sequencing: The amplification products of circCGNL1 in a T vector were sent to Sangon (Shanghai, China) for Sanger sequencing analysis. The primers were synthesized and designed to verify the back splice junction of circCGNL1. The primer sequences used in this study are listed in Supplementary Table 1.

### Transfection

PANC-1 and BxPC-3 cells were transfected in six-well plates with Lipofectamine 3000 (L3000075, Invitrogen, Carlsbad, CA, USA). NUDT4 and RUNX2 short-hairpin RNAs (shRNAs) and negative control (NC) shRNAs were purchased from GenePharma (Shanghai, China). HDAC4, RUNX2, and GAMT overexpression plasmids were separately generated by inserting full-length HDAC4, RUNX2, and GAMT cDNA sequences into the pcDNA3.1 vector, with the empty vector serving as an NC. The circCGNL1 sequence was cloned into pcDNA3.1( +) circRNA Mini Vector (LM8145, LMAI Bio, Shanghai, China) and used to construct stable circCGNL1-overexpressing cell lines. The sequences used in this research are listed in Supplementary Table 1. After the transfected cells grew to 80% density, neomycin (60207ES25, Yeasen, Shanghai, China) was added to screen cells expressing the target genes at a dose of 600ug/ml and the surviving cells were continuously cultured with 200ug/ml neomycin concentration.

### Proliferation assays

Cell Counting Kit-8 (CCK-8) assay: transfectants were seeded in 96-well plates at 2,000 cells/well in 100 µl medium. Subsequently, the cells were treated with 10 μl CCK-8 reagent (M4839, ABMOLE, USA) at different time points. Optical density (ODs) was measured at 450 nm wavelength using a microplate reader (51119770DP, ThermoFisher). EdU experiments were performed using a Cell-Light EdU DNA Cell Proliferation Kit (C0075, Beyotime). Following the manufacturer’s protocol, PANC-1/BxPC-3 cells were incubated with 50 mM EdU solution for 2 h and immobilized in paraformaldehyde (4%). After that, the cells were permeabilized with 0.3% Triton (9036–19-5, Sigma-Aldrich) for 10 min and then sequentially stained with Alexa Fluor 555 azide. Subsequently, three non-overlapped visions with evenly distributed cells were selected randomly for capturing at 200 × magnification by a fluorescence microscope (DMi8, Leica, Germany) at same exposure time. The cell counting was performed by ImageJ 1.52, the threshold was set as 10 in dark background model, and analyze particles were set as 30-infinity. Proliferation assays were independently repeated three times.

### Apoptosis assays

Terminal deoxynucleotidyl transferase dUTP nick end labeling (TUNEL): TUNEL Apoptosis Assay Kit (C1088, Beyotime) was used to determine apoptosis. Cells (1 × 10^4^) were seeded in 96-well plates and treated with 4% paraformaldehyde and 0.1% Triton (9036–19-5, Sigma-Aldrich). Based on the manufacturer’s protocol, TUNEL reagent (50 μl/well) was used to incubated with cells for 60 min at 37ºC. The cells were then treated with DAPI (D9542, Sigma-Aldrich) for nuclear staining. Image acquisition was performed in three non-overlapped visions with evenly distributed cells at 200 × magnification using the fluorescence microscope (DMi8, Leica) with same exposure time. The cell counting was performed by ImageJ (Dark background, Threshold 10, Analyze particles 30-infinity). Flow cytometry experiments: Cells in six-well plates were washed with PBS (C0221A, Beyotime) and fixed with ice-cold ethanol (45726, Sigma-Aldrich). Then, the cells were stained with propidium iodide (ST512, Beyotime) and fluorescein isothiocyanate-conjugated annexin V (C1062L, Beyotime). Finally, apoptosis was detected using flow cytometry (LSR, BD, Biosciences). DAPI staining assay: 500 circCGNL1-overexpressed or vector pcDNA3.1 cells were seeded in six-well plates and cultured for 48 h. The cells were rinsed with PBS for 5 min and stained with DAPI. Results were observed at 359 nm excitation light under the fluorescence microscope at 1000 × magnification. Apoptotic cells showed nucleus condensation, fragmentation, deep staining or nucleus shrinkage. The cell fluorescence intensity rate was defined as total fluorescence intensity of apoptotic cells versus total fluorescence intensity of normal cells. ImageJ-ROI manager was used to measure the fluorescence intensity in three visions. Transmission electron microscopy (TEM) assay: circCGNL1 overexpressed BxPC-3 and PANC-1 cells were prepared following the transfection method. Cell samples were collected and fixed overnight at 4 °C in 2.5% glutaraldehyde solution, then were embedded with acetone and embedding agent. The embedded samples were stained with lead citrate solution and uranyl acetate 50% ethanol saturated solution for 15 min. Then, TEM (Tecnai G2 Spirit, FEI, USA) was used to observe the morphological changes of circCGNL1-overexpressed or pcDNA3.1 control cells. Apoptosis assays were independently repeated three times.

### Western blot (WB) analysis

Proteins were isolated using a protein extraction kit (PROTTOT-1KT, Sigma-Aldrich) and quantitated using Bradford Protein Assay Kit (P0012, Beyotime). The proteins (30 ug each lane) were separated using SDS-PAGE gels (P0690, Beyotime) and transferred to PVDF membranes (IPVH00010, Millipore), which were blocked with TBST solution containing 3% BSA (36104ES, Yeasen, China) for 2 h. The membranes were then incubated at 4 °C overnight with appropriate primary antibodies, which were diluted by TBST solution (3% BSA). An HRP-conjugated secondary antibody and enhanced chemiluminescence luminescent solution (P0018S, Beyotime) were used to detect the protein bands. The bands gray value was measured by ImageJ. The primary and secondary antibodies used in this study are listed in Supplementary Table 2.

### Subcellular fractionation and fluorescence in situ hybridization (FISH) assay

Cytoplasmic and Nuclear RNA Purification Kit (Norgen, Ontario, Canada) was used to isolate and purify cytoplasmic and nuclear RNA. The cytoplasmic and nuclear circCGNL1 contents were analyzed using qRT-PCR, with ACTB or U6 expression serving as internal controls. Cellular circCGNL1 distribution was detected using Ribo™ Fluorescent In Situ Hybridization Kit (C10910, RiboBio). The fluorescent-labelled circCGNL1 probe was synthesized by RiboBio and the sequence was listed in Supplementary Table 1. DAPI was used for nuclear staining. Images were acquired with an LSM880 NLO (2 + 1 with BIG) confocal microscope system (Carl Zeiss). Fluorescence intensity rate was defined as the average fluorescence intensity of nuclear or cytoplasm versus the intensity of the whole cells. Fluorescence intensity was measured by ImageJ (Dark background, Threshold 20) in three visions.

### Immunofluorescence (IF) staining

For the detection of NUDT4 and HDAC4, transfected PANC-1 and BxPC-3 cells were incubated in the coverslips of six-well plates at 37 °C overnight. The cells were washed with PBS twice, fixed with 4% formaldehyde and then permeabilized with 0.5% Triton (9036–19-5, Sigma-Aldrich). The treated cells were incubated with primary antibody overnight at 4 °C and then with secondary antibody. Sample mounting and nuclear staining were performed using ProLong™ Gold Antifade Mountant with DNA Stain DAPI (P36935, ThermoFisher). The samples stained only with secondary antibody were set as negative control. IF images were obtained using a fluorescence microscope (DMi8, Leica, Germany). Fluorescence intensity analysis was the same with FISH assay. The primary and secondary antibodies are listed in Supplementary Table 2.

### Co-immunoprecipitation (Co-IP) and GST pulldown assays

Cell lysates were obtained via incubation with IP lysis buffer and incubated with appropriate antibodies at 4 °C and rotation at a constant speed. Normal IgG served as a control antibody. Next, the mixture was incubated with beads for 2 h, after which the beads were washed with IP lysis buffer, and immunoprecipitated proteins were eluted for immunoblotting. For the GST pulldown experiments, GST-NUDT4 or GST-HDAC4 was purified using GSTrap FF (GE Healthcare). Total cell lysates were incubated with GST beads (Biosciences, Sweden) overnight at 4 °C and rinsed with washing buffer. Finally, proteins were visualized using immunoblotting.

### RIP assay

RIP assay was conducted using Imprint® RNA Immunoprecipitation Kit (RIP-12RXN, Sigma-Aldrich). Cells (~ 1 × 10^7^) were lysed in 1 ml RIP lysis buffer containing protease and RNase inhibitors. The cell lysates were incubated with IgG (ab6789, Abcam) and anti-NUDT4 antibody-coated beads (Millipore) and rotated at 4 °C overnight. The immunoprecipitated RNAs were then treated with proteinase K buffer, extracted using RNeasy MinElute Cleanup Kit (74204, Qiagen, Valencia, CA, USA), and reverse transcribed to cDNA. Subsequently, the RNA levels of target genes were evaluated using qRT-PCR.

### Chromatin immunoprecipitation (ChIP)

A ChIP kit (17–295, Millipore, Bedford, MA, USA) was used for the ChIP assay. Briefly, the cells were fixed, DNA–protein cross-links were formed, the DNA was randomly fragmented using ultrasonication, and DNA–protein complexes were immunoprecipitated with RUNX2 or IgG antibody (control) using magnetic beads.

### RNA pulldown assay

Pierce™ Magnetic RNA–Protein Pull-Down Kit (20164, ThermoFisher) was used in the RNA pulldown assay, based on the manufacturer’s protocol. The biotinylated circCGNL1 or negative control probe was incubated with cell lysates at room temperature for 60 min and then magnetic beads were added for 45 min at 4 °C with rotation. Subsequently, the RNA–protein complexes were washed with wash buffer twice and incubated with elution buffer for 30 min at 37 °C with agitation. Remove the beads and harvested the supernatant for downstream analysis. The pulled down proteins were run on SDS-PAGE gels, and then gels were stained by silver staining (P0017S, Beyotime), according to the manufacturer’s instructions. Pulldown proteins were also sent to Wininnovate bio (Shenzhen, China) for mass spectrographic analysis. The circCGNL1 and negative control probe sequences are listed in Supplementary Table 1.

### Luciferase reporter analysis

Wild-type and mutated binding sites in the GAMT promoter were cloned into the pGL3 vector (E1751, Promega, Madison, WI, USA), and cells were co-transfected with either pcDNA3.1-RUNX2 plasmids or empty vector. After 48 h, luciferase activity was detected using Luciferase Reporter Assay System according to the manufacturer’s instructions (Promega).

### Immunoprecipitation (IP)-WB analysis

The cells were harvested and rinsed with ice-cold PBS twice and then resuspended in 1X lysis buffer (20–188, Millipore, Bedford, MA, USA) for 30 min at 4 °C. Harvested the supernatant after centrifugation at 12000 rpm for 30 min. Pierce™ Protein A/G Magnetic Beads (88802, ThermoFisher) were used in the IP-WB assays, according to the manufacturer’s protocol. Magnetic Beads were rinsed with PBS and 0.5% Triton (9036–19-5, Sigma-Aldrich) and then incubated overnight with appropriate antibodies at 4 °C. Subsequently, the cell lysates were then incubated with the antibody-beads complex for 60 min at 4 °C with agitation. Finally, the bound proteins were eluted by PBS and 0.5% Triton and probed with antibodies against specific proteins.

### In vivo tumor growth assay

Male, 6–8-week-old BALB/c mice (Shanghai Experimental Animal Research Center) were randomly divided into two groups (five mice/group). PANC-1 cells were transfected with either pcDNA3.1-circCGNL1 or empty pcDNA3.1 vector and cultured for 48 h. Subsequently, 2 × 10^6^ cells were injected subcutaneously into each nude mouse (2 × 10^6^ cells/0.1 ml). After 7 days, the tumor volumes were measured every 3 days up to 27 days post-injection. The excised tumors were harvested for analysis after sacrificing the mice via cervical dislocation. The experiments were approved by the Animal Research Ethics Committee of Heping Hospital Affiliated of Changzhi Medical College (DW2023089) and the Institutional Animal Care and Use Committee (IACUC) of Nanjing Medical University (IACUC-2302020).

### Immunohistochemistry (IHC)

After the tissues from the in vivo tumor-formation assays were deparaffinized with xylene and rehydrated with ethanol, the samples were incubated with 3% H_2_O_2_ for 5 min to block endogenous peroxidase activity. Then antigen retrieval was performed via sodium citrate buffer (pH 6.0) for 20 min at 95 °C and samples were blocked with 5% normal goat serum for 10 min at 20 °C. The sections were incubated overnight with primary antibodies against Ki67, cleaved caspase-3, and PCNA at 4 °C and then with a secondary antibody for 30 min. The tissue sections were captured, and protein levels were calculated as positive cells/total cells by ImageJ (RGB stack: Blue, Threshold: auto, Dark background). All the primary and secondary antibodies used in this study are listed in Supplementary Table 2.

### Bioinformatic analysis

Raw transcriptome data (patients with PC and healthy donors) were downloaded from The Cancer Genome Atlas (TCGA), and genotype-tissue expression (GTEx) data were downloaded from the UCSC Xena database (http://xena.ucsc.edu/). TCGA data were normalized by performing log_2_ (x + 1) transformation, which enabled comparison with the GTEx data. We analyzed 178 PC samples and 169 control samples. Differentially expressed genes (DE-genes) between PC and normal pancreas tissues were identified using the “limma” R package based on a log_2_ fold-change (FC) of > 1 and an adjusted *p* value of < 0.01 [27]. Online bioinformatic tools: Online stool HitPredict (http://www.hitpredict.org/) and BioGRID (https://thebiogrid.org/) were used to obtain the proteins that may bind to NUDT4 based on the website instruction (keyword ‘NUDT4’). GEPIA (http://gepia.cancer-pku.cn/index.html) was used to analyze the expression of nine selected genes in normal and tumor tissues (|log_2_FC|> 1, *p* value < 0.01; TCGA data were normalized with GTEx data).

### Statistical analyses

Statistical analyses (one-way ANOVA or Student’s t-test) were performed with GraphPad Prism 6 (GraphPad Software, Inc., La Jolla, CA, USA). In this study, T-test and single/bidirectional ANOVA were used in both qPCR and cell function tests to analyze the differences in cell function between the experimental groups. Survival curves were plotted based on the Kaplan–Meier method. Univariate and multivariate Cox regression analysis was performed by SPSS version 20.0 (SPSS Inc., Chicago, IL, USA). Baseline variables showed a univariate relationship with OS were selected into multivariate Cox regression model. Quantitative data are presented as the means ± SDs from three independent experiments. Statistical significance was set at *P* < 0.05.

## Results

### Characteristics of circCGNL1 in PC

**Fig. 1:**
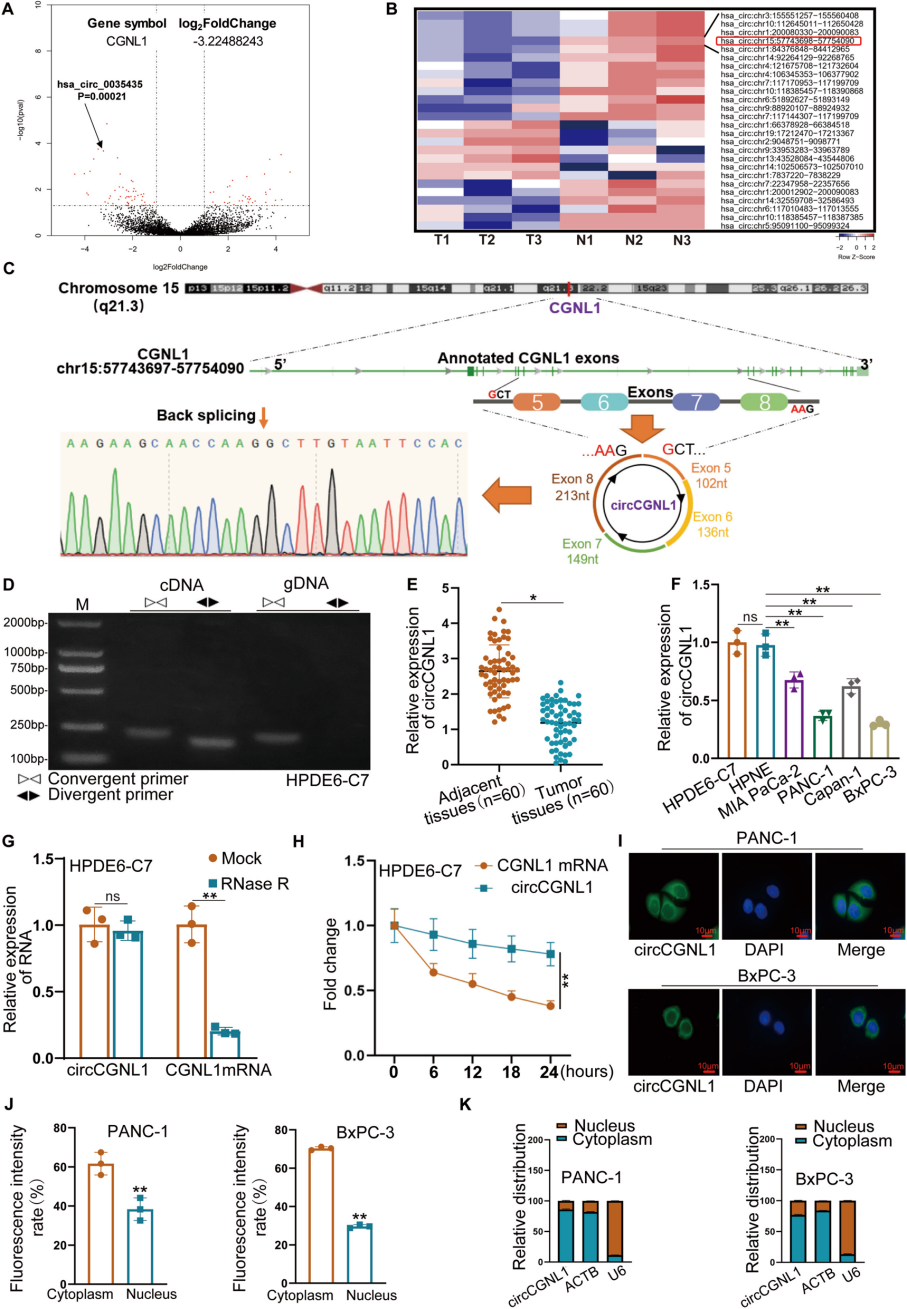
Identification and characteristics of circCGNL1 in PC cells and tissues. **A**, **B** Volcano plots [30] and a heatmap indicated that circCGNL1 (has_circ_0035435; chromosome 15, nt positions 57743698-57754090) was expressed at lower levels in PC tissues (log_2_FC = -3.22) than in matched normal tissues. **C** Schematic illustration of circCGNL1 formation via circularization of exons 5–8 of a CGNL1 mRNA transcript. The back splicing site was validated using Sanger sequencing. **D** Agarose gel electrophoresis was performed to detect the cyclization of circCGNL1 in HPDE6-C7 cells. **E**, **F** qRT-PCR assays were performed to detect circCGNL1 expression in PC tissues and different cell lines (MIA PaCa-2, PANC-1, Capan-1, BxPC-3, HPDE6-C7 and HPNE). **G** qRT-PCR analysis showing the expression levels of circCGNL1 and CGNL1 mRNA in HPDE6-C7 cells subjected to RNase R treatment. **H** RNA-expression levels of circCGNL1 and CGNL1 in HPDE6-C7 cells analyzed using qRT-PCR after treatment for 24 h with actinomycin D (2 μg/mL). **I-K** FISH and Subcellular-fractionation assays were used to determine subcellular localization of circCGNL1 in PANC-1 and BxPC-3 cells. Fluorescence intensity of nucleus and cytoplasm was measured via ImageJ. Scale bar = 10 μm. ACTB (cytoplasm) and U6 (nucleus) were served as internal controls. ns: no significance, *p < 0.05, **p < 0.01

Previously, we identified PC-associated circRNA transcripts using three pairs of pancreatic ductal carcinoma and adjacent normal tissues [28, 29]. Here, we found that circCGNL1 (hsa_circ_0035435, chr15:57743698–57754090) was downregulated in the three PC tissue samples (Fig. 1A,B). Based on the circBase annotation database, circCGNL1 is derived from exon 5 (102 nt), exon 6 (136 nt), exon 7 (149 nt), and exon 8 (213 nt) of *CGNL1*. Sanger sequencing of circCGNL1 cDNA verified back splicing (Fig. 1C). Divergent and convergent primers were designed to amplify the circular and linear forms of CGNL1, respectively. Agarose gel electrophoresis showed that circCGNL1 could only be amplified from cDNA, ruling out the possibility of genomic rearrangements and trans-splicing (Fig. 1D). Moreover, circCGNL1 expression was significantly lower in the 60 PC tissues than in the paired adjacent normal tissues (Fig. 1E). circCGNL1 expression was also lower in the four PC cell lines (MIA PaCa-2, PANC-1, Capan-1, and BxPc-3) than in the normal pancreatic cell lines (HPDE6-C7 and HPNE) (Fig. 1F) and particularly low in PANC-1 and BxPC-3 cells. Hence, we selected PANC-1 and BxPC-3 cell lines to detect the function and mechanism of circCGNL1. RNase R and actinomycin D-treatment assays showed that circCGNL1 was more stable than linear CGNL1, when treated with RNase R or actinomycin D (Fig. 1G,H). To explore the subcellular localization and potential function method of circCGNL1, FISH and cytoplasmic fractionation assays were performed. The results showed circCGNL1 was expressed in both the nucleus and cytoplasm, but mainly in the cytoplasm, which indicated circCGNL1 might interact with miRNAs or RNA Binding Proteins in PC (Fig. 1I-K supplementary Fig. 1A,B). In summary, circCGNL1 was expressed at low levels in PC and was predominantly localized in the cytoplasm of PC cell lines.

### CircCGNL1 represses cell proliferation and promotes PC cell apoptosis

**Fig. 2:**
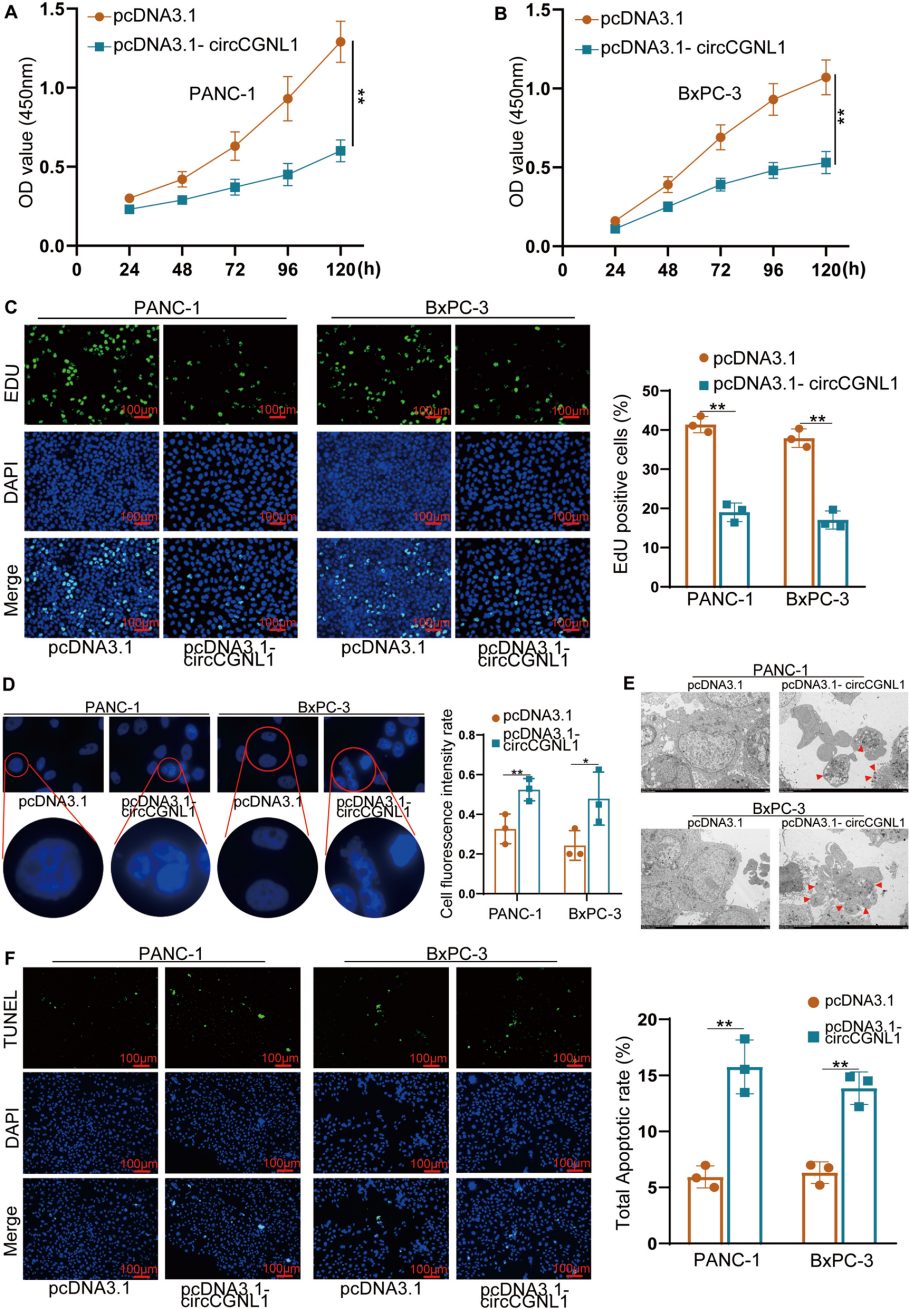
circCGNL1 represses PC cell proliferation and promotes PC cell apoptosis. **A**, **B** CCK-8 assays were performed to detect the viability of PANC-1 and BxPC-3 cells transfected with either pcDNA3.1 or pcDNA3.1-circCGNL1. **C** EdU-incorporation assays showing that circCGNL1 overexpression significantly reduced the proliferation of PANC-1 and BxPC-3 cells. Scale bar = 100 μm. **D**, **E** DAPI staining assay was applied to measure PC cell apoptosis (**D**) and the cell nuclear chromatin morphological changes was observed via TEM (**E**). The red arrows showed the forming of apoptotic bodies. **F** TUNEL assays showing that apoptosis was significantly higher in PANC-1 and BxPC-3 cells transfected with pcDNA3.1-circCGNL1 than in those transfected with empty pcDNA3.1 vector. Scale bar = 100 μm. *p < 0.05, **p < 0.01

qRT-PCR analysis indicated that circCGNL1 expression was elevated in PANC-1 and BxPC-3 cells transfected with the pcDNA3.1-circCGNL1 plasmid (Supplementary Fig. 1C). CCK-8 assays were performed to study the effect of circCGNL1 upregulation on cell viability. pcDNA3.1-circCGNL1 PANC-1 and BxPC-3 transfectants showed substantially lower OD values than pcDNA3.1 transfectants (Fig. 2A,B), suggesting that circCGNL1 overexpression inhibited viability, which was further supported by our EdU assay results (Fig. 2C). DAPI staining assay was applied to detect tumor cell apoptosis. The results demonstrated that more cell apoptosis was in circCGNL1 overexpression group and cells morphological changes were observed in the nuclear chromatin including nucleus collapse and apoptotic body formation by TEM (Fig. 2D,E). In agreement, the proportion of TUNEL-positive cells was significantly higher with pcDNA3.1-circCGNL1 transfectants than with control transfectants (Fig. 2F). Flow cytometric analysis also showed circCGNL1 overexpression promoted PANC-1 and BxPC-3 cell apoptosis (Supplementary Fig. 1D-F). Mia-PaCa-2 and Capan-1 cells, which have relatively high circCGNL1 expression, were further used to perform TUNEL and CCK8 experiments after knocking down circCGNL1. We found circCGNL1 knockdown inhibited apoptosis and promote proliferation significantly in Mia-PaCa-2 and Capan-1 cells (Supplementary Fig. 1G,H). Combined, these data indicate that circCGNL1 promoted cell apoptosis and repressed cell proliferation in PC.

### circCGNL1 interacts with NUDT4 protein in PC cells

**Fig. 3:**
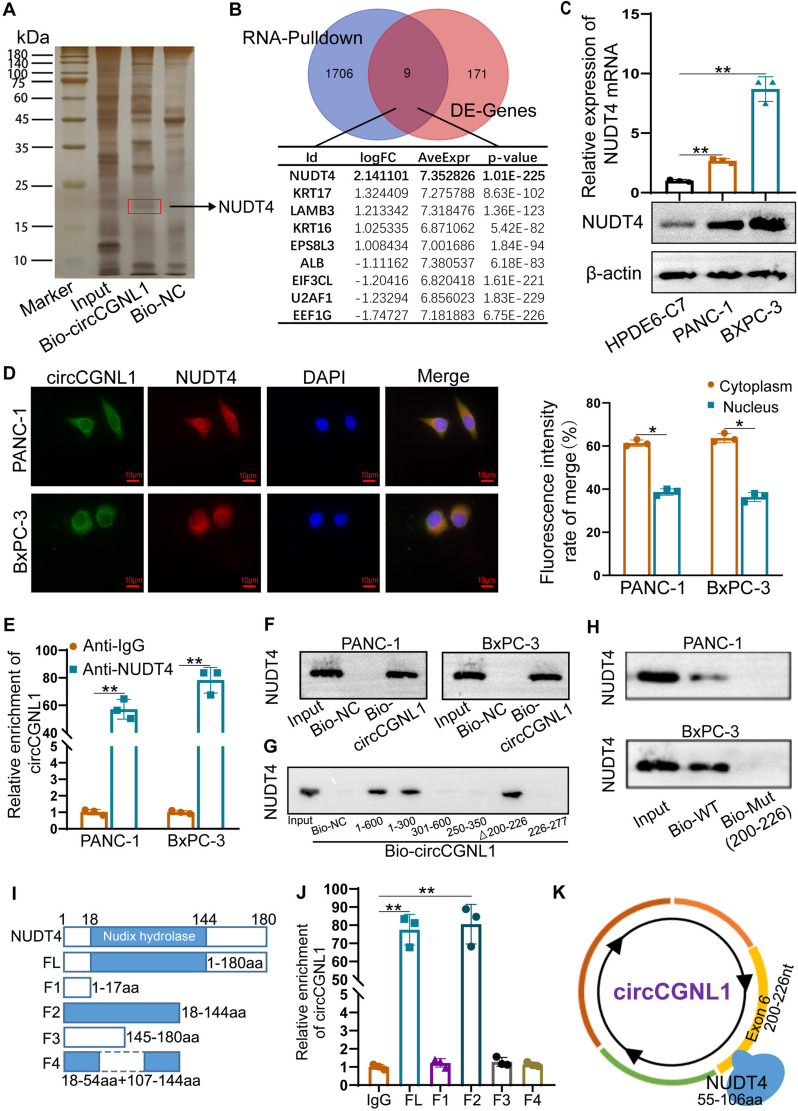
circCGNL1 physically interacts with NUDT4 protein in PC cells. **A** Biotinylated circCGNL1 probe (Bio-circCGNL1) and biotinylated antisense circCGNL1 probe (Bio-NC) were transcribed in vitro and incubated with protein extracts from PANC-1 cells for RNA pulldown experiments. Pulled-down proteins were harvested for silver staining, and a specific band was observed between 15 and 25 kDa (arrow). **B** MS and DEG analyses were performed to identify candidate proteins that could interact with circCGNL1. Nine overlapping potential binding proteins were identified and are shown. **C** qRT-PCR and WB assays were conducted to detect mRNA and protein expression levels of NUDT4 in PANC-1 and BxPC-3 cells. **D** FISH-IF assays showing that NUDT4 colocalized with circCGNL1 in PANC-1 and BxPC-3 cells. Scale bar = 10 μm. **E** RIP experiments were performed using antibodies against NUDT4 and IgG, and qRT-PCR was performed to detect circCGNL1 in PANC-1 and BxPC-3 cells. β-actin expression was detected as an internal control. **F** RNA pulldown assays further confirmed the binding between NUDT4 and circCGNL1. **G** Immunoblotting for NUDT4 pulled down with the circCGNL1 antisense control and different circCGNL1 truncation variants (nt 1–600, 1–300, 301–600, 250–350, 200–226, and 226–277). **H** Wild type (WT) and 200-226nt mutated (Mut) circCGNL1 probes were used to performed RNA pulldown assay. **I**, **J** NUDT4 protein was truncated into different fragments, including aa 1–17, 18–144 (the nudix hydrolase domain of NUDT4), 145–180, 18–54, or 107–144 (**I**). RIP assays were performed with the different truncated NUDT4 fragments in 293 T cells (**J**). **K** Graphic illustration of the interaction sites between circCGNL1 exon 6 and NUDT4. *p < 0.05, **p < 0.01

CircRNAs exhibited their biological functions via multiple methods including miRNA sponge and RNA bind protein (RBP). The function of circRNA as miRNA sponge requires binding with AGO2 to form a circRNA-AGO2-miRNA complex. Anti-Ago2 and anti-lgG were used to perform RIP experiment, which showing Ago2 couldn’t enrich circCGNL1 (Supplementary Fig. 2A). Hence, miRNA sponge may not be main function way for circCGNL1 in PC. RNA-pulldown and mass spectrometry analysis were further performed to determine the RBPs interacting with circCGNL1. The results revealed 1,715 potential circCGNL1-binding proteins in PC cell lysates (Supplementary Table 3). Transcriptome data of 178 PC samples and 169 control pancreatic tissue samples (TCGA and GTEx databases) were studied via bioinformatic analysis, revealing 180 DE-genes (Supplementary Table 4 and Supplementary Fig. 2B), of which 70 were upregulated and 110 were downregulated (Supplementary Fig. 3A). Silver staining and overlapping analysis between RNA pulldown and DE-genes results revealed nine potential circCGNL1-interacting partners, among which NUDT4 was the most differentially expressed and strongly upregulated in PC (Fig. 3A, B). All nine potential genes were verified using the GEPIA database, and the NUDT4 expression trend was consistent with the bioinformatic analysis results (Supplementary Fig. 3B–J). Hence, NUDT4, a dephosphorylase, was selected as the potential target of circCGNL1.

qRT-PCR and WB assays indicated that NUDT4 was expressed at higher levels in PANC-1 and BxPC-3 cells (Fig. 3C). Furthermore, the Hum-mPLoc website (http://www.csbio.sjtu.edu.cn/bioinf/hum-multi-2/) predicted that NUDT4 is mainly located in the cytoplasm (Supplementary Fig. 4A). By qRT-PCR and WB assays, we found circCGNL1 overexpression did not affect NUDT4 mRNA and protein expression (Supplementary Fig. 4B,C). Next, FISH-IF assays were performed to test the hypothesis that circCGNL1 recruits NUDT4 to modify NUDT4-mediated regulation of downstream target proteins. We found circCGNL1 and NUDT4 to be colocalized in the cytoplasm of PANC-1 and BxPC-3 cells mainly (Fig. 3D). RIP assays revealed that circCGNL1 was significantly enriched in the anti-NUDT4 group (Fig. 3E), suggesting that circCGNL1 bound to NUDT4 directly. This was confirmed by RNA pulldown assays, showing NUDT4 was pulled down with a biotinylated circCGNL1 probe (Fig. 3F). Moreover, the catRAPID website (tartaglialab.com) predicted a potential binding site between circCGNL1 and NUDT4 (Supplementary Fig. 4D). Then, RNA pulldown assays were designed using truncated circCGNL1s. The fragment comprising nucleotides (nt) 200–226 of circCGNL1 could pulldown NUDT4, which was also confirmed in 200–226 mutated circCGNL1 probe (Fig. 3G,H). Hence, we further tested full-length NUDT4 and different peptides, including amino acids (aa) 1–17, 18–144 (Nudix hydrolase area), 145–180 and 18–54 together with 107–144 in RIP experiments (Fig. 3I). NUDT4 hydrolase domain (18–144aa) was enriched for circCGNL1 from 293 T cell lysates, however, the 18–54 together with 107–144 fragments were not (Fig. 3J). Taken together, circCGNL1 interacted with NUDT4, and 200-226nt on exon6 of circCGNL1 was mainly responsible for the interaction with 55-106aa on NUDT4 Nudix hydrolase area (Fig. 3K). Furthermore, NUDT4 expression was knocked down in PANC-1 and BxPC-3 cells using plasmids encoding NUDT4 short-hairpin RNAs (sh-NUDT4 plasmids). qRT-PCR showed that the interference effects of sh1-NUDT4 and sh2-NUDT4 were the strongest (Supplementary Fig. 5A). Thus, sh1-NUDT4 and sh2-NUDT4 were selected for subsequent functional assays. CCK-8 and EdU-based proliferation assays showed that PANC-1 and BxPC-3 cell growth was inhibited upon NUDT4 downregulation (Supplementary Fig. 5B-D). TUNEL and flow cytometric assays showed NUDT4 depletion accelerated apoptosis (Supplementary Fig. 5E,F). Moreover, more apoptotic tumor cells occurred in NUDT4 knockdown groups in DAPI staining assay and apoptotic body were observed via TEM (Supplementary Fig. 5G,H). In summary, these data demonstrated that circCGNL1 directly interacted with the NUDT4-Nudix hydrolase domain and that NUDT4 exerted as an oncogene in PC.

### NUDT4 mediates HDAC4 dephosphorylation

**Fig. 4:**
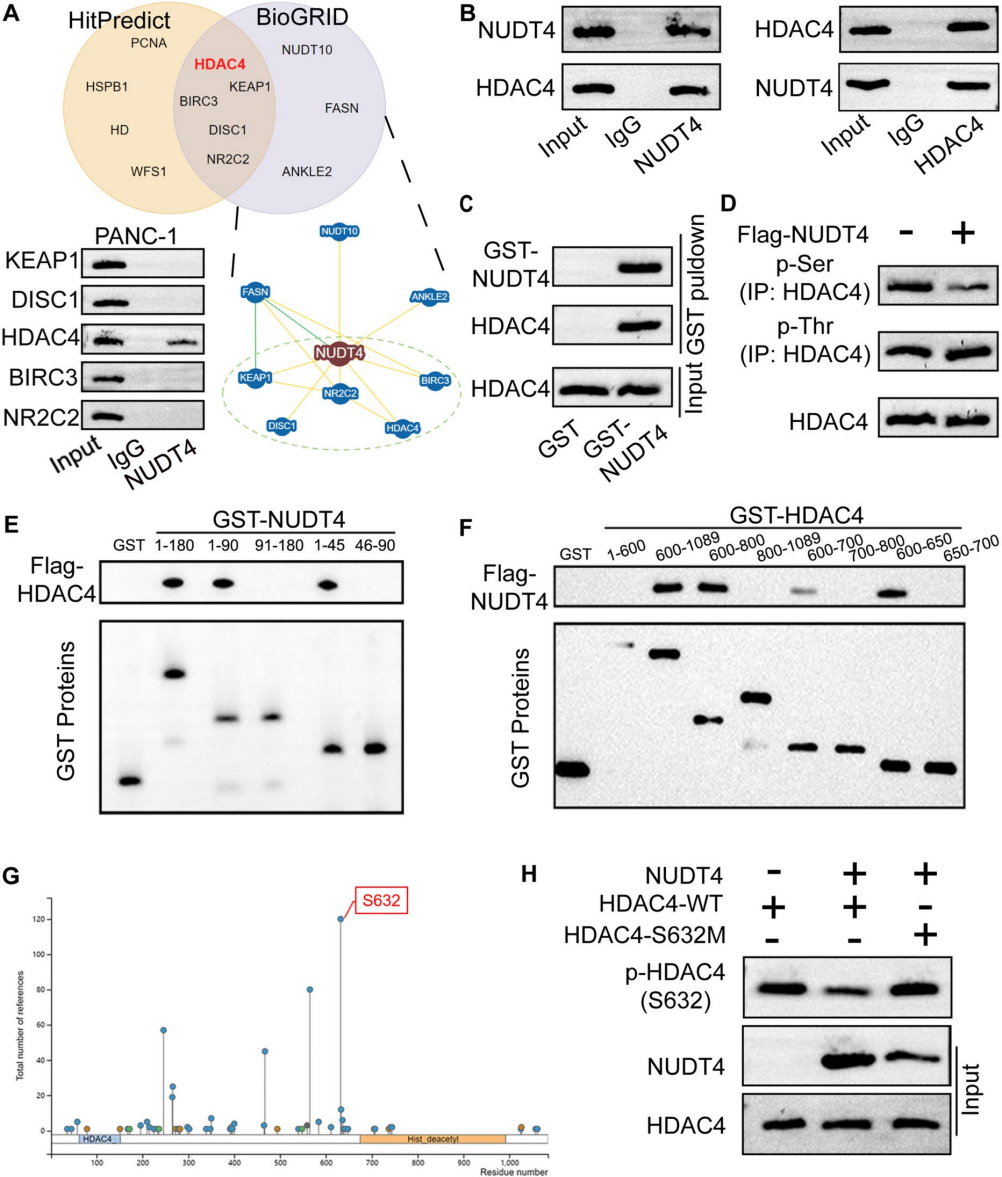
NUDT4 regulates dephosphorylation of HDAC4 protein. **A** The online HitPredict and BioGRID bioinformatic tools were used to predict candidate NUDT4-interacting proteins, and candidate proteins identified with both tools were screened using Co-IP assays. **B**, **C** GST pulldown and Co-IP assays confirmed the physical interaction between NUDT4 and HDAC4 in 293 T cells. Anti-lgG and anti-NUDT4 antibodies were used in the Co-IP experiments (**B**), and GST-NUDT4 was used for the GST pulldown assay (**C**). **D** IP-WB assays illustrated that NUDT4 inhibited serine phosphorylation (p-Ser) rather than threonine phosphorylation (p-Thr) on HDAC4. **E**, **F** GST pulldown assays were performed to analyze the binding of NUDT4 fragments (aa 1–180, 1–90, 91–180, 1–45, or 46–90) with HDAC4 fragments (aa 1–600, 600–1089, 600–800, 800–1089, 600–700, 700–800, 600–650, or 650–700) in 293 T cells, which showed that aa 1–45 of NUDT4 interacted with aa 600–650 of HDAC4. **G** PhosphoSitePlus online stool was used to predict the phosphorylation sites of HDAC4. **H** IP-WB experiments indicated that NUDT4 could dephosphorylate WT HDAC4 at S632, and this dephosphorylation was hindered when S632 was mutated (HDAC4-S632M)

NUDT4 functions as a phosphatase, by regulating the phosphorylation level of the downstream interacting proteins. We predicted candidate NUDT4-interacting proteins using HitPredict (http://www.hitpredict.org/) and BioGRID (https://thebiogrid.org/). By combining both sets of predictions, we identified five potential downstream proteins of NUDT4, including KEAP1, DISC1, HDAC4, BIRC3, and NR2C2, of which only HDAC4 could be detected in the NUDT4 eluent group in Co-IP experiments (Fig. 4A). Moreover, Co-IP assays performed in 293 T cells with HDAC4 or NUDT4 as the bait protein confirmed that NUDT4 could interact with HDAC4 (Fig. 4B). GST pulldown assays further proved that HDAC4 directly interacted with NUDT4 (Fig. 4C). IP-WB assays showed that NUDT4 promoted serine dephosphorylation of HDAC4 (Fig. 4D). Hence, we performed GST pulldown assays to identify binding sites important for NUDT4–HDAC4 fragment interactions in 293 T cells. The results showed NUDT4 aa 1–45 bound the aa 600–650 fragment of HDAC4 (Fig. 4E,F). In addition, predictions by PhosphoSitePlus (https://www.phosphosite.org/) showed a high score for a potential phosphorylation site at HDAC4 residue S632 (Fig. 4G). Therefore, we constructed HDAC4-expression vectors containing a mutant S632 codon or the wild type (WT) S632 codon and used the vectors for GST pulldown experiments. NUDT4 promoted WT HDAC4 dephosphorylation without affecting dephosphorylation of the HDAC4-S632M variant (Fig. 4H). These results indicate that NUDT4 interacted with HDAC4 to mediate the dephosphorylation of S632 in HDAC4.

### CircCGNL1 promotes HDAC4 dephosphorylation by recruiting NUDT4

**Fig. 5:**
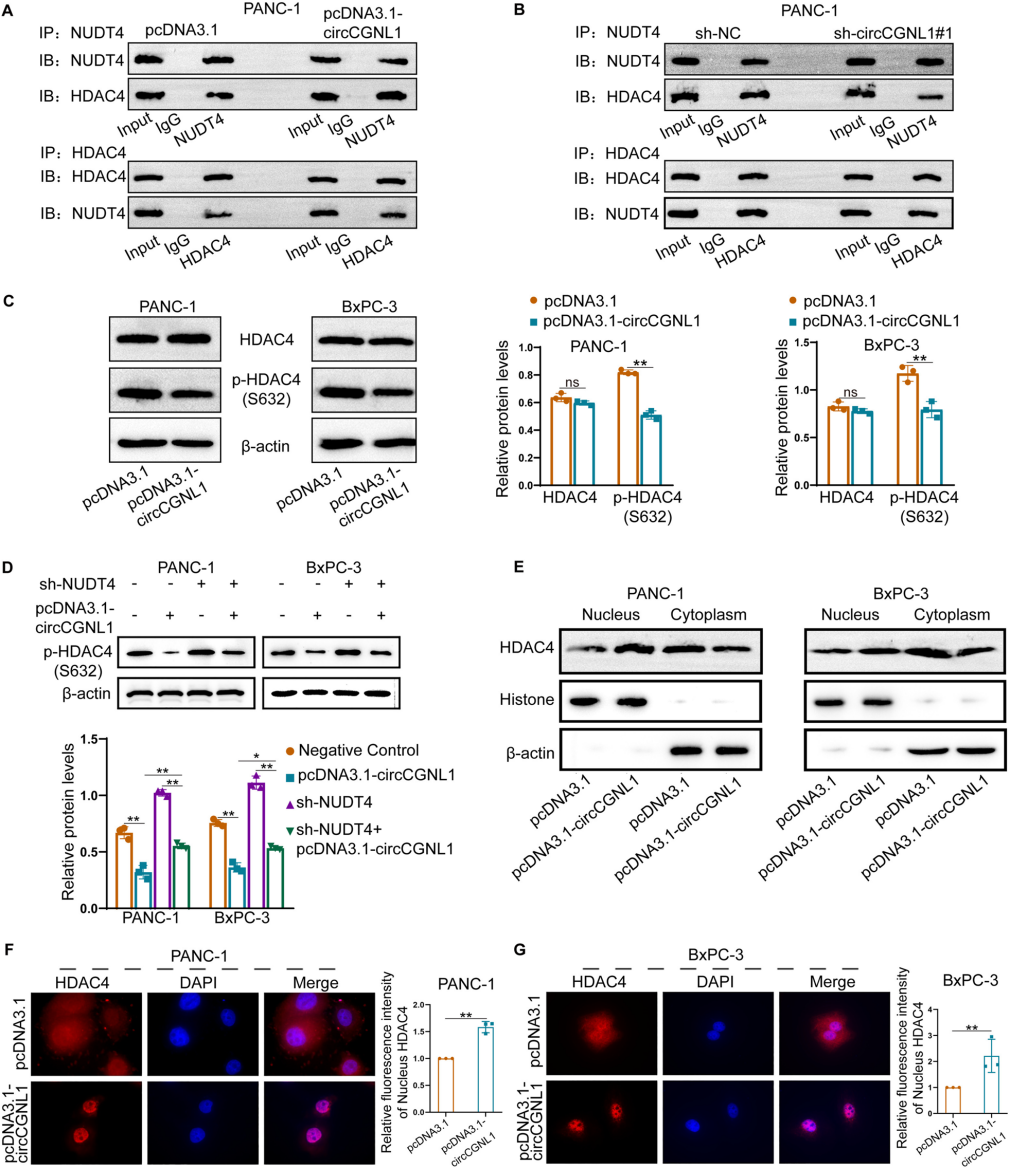
circCGNL1 enhances HDAC4 dephosphorylation by interacting with NUDT4. **A**, **B** We overexpressed or knocked down circCGNL1 in PANC-1 cells and performed Co-IP assays with NUDT4 or HDAC4 as bait proteins. Immunoblotting showed that the combination of NUDT4 and HDAC4 was increased by circCGNL1. **C** WB analysis indicated that HDAC4 serine phosphorylation decreased significantly following circCGNL1 overexpression. **D** Rescue experiments showed that silencing NUDT4 reversed the dephosphorylation effect of circCGNL1 on HDAC4 (S632). **E–G** The cytoplasmic and nuclear HDAC4-expression levels in PC cells transfected with pcDNA3.1 (empty vector) or pcDNA3.1-circCGNL1 were detected by WB (**E**) and IF assays (**F**, **G**). Relative fluorescence intensity of nucleus HDAC4 was the fluorescence intensity of nuclear HDAC4 in circCGNL1 overexpressing group versus that in empty vector group. The fluorescence intensity of Nucleus HDAC4 was measured by ImageJ. ns: no significance, *p < 0.05, **p < 0.01

Previous experimental results showed that circCGNL1 could interact with NUDT4, and NUDT4 could promote the dephosphorylation of HDAC4 (S632). Therefore, we explored the regulation of circCGNL1 on HDAC4 phosphorylation. Co-IP assays showed that circCGNL1 significantly strengthened the NUDT4–HDAC4 interaction in PANC-1 and BxPC-3 cells (Fig. 5A,B, Supplementary Fig. 6A). PANC-1 and BxPC-3 cells transfected with pcDNA3.1-circCGNL1 have lower HDAC4 S632 phosphorylation level, however, without affecting total HDAC4 expression (Fig. 5C). WB assays showed that silencing NUDT4 expression partly rescued the inhibition of HDAC4 phosphorylation by circCGNL1 (Fig. 5D). Phosphorylation of HDAC4 at S632 is crucial for nuclear HDAC4 translocation. Therefore, we explored the effect of circCGNL1 on nuclear HDAC4 translocation in PC cells. WB and IF assays showed that HDAC4 could be detected in cytoplasm, however, circCGNL1 overexpression substantially increased nuclear HDAC4 levels (Fig. 5E-G). These results demonstrated that circCGNL1 facilitated S632 dephosphorylation and the nuclear transfer of HDAC4 by interacting with NUDT4.

### CircCGNL1 regulates RUNX2 acetylation and degradation via HDAC4

**Fig. 6:**
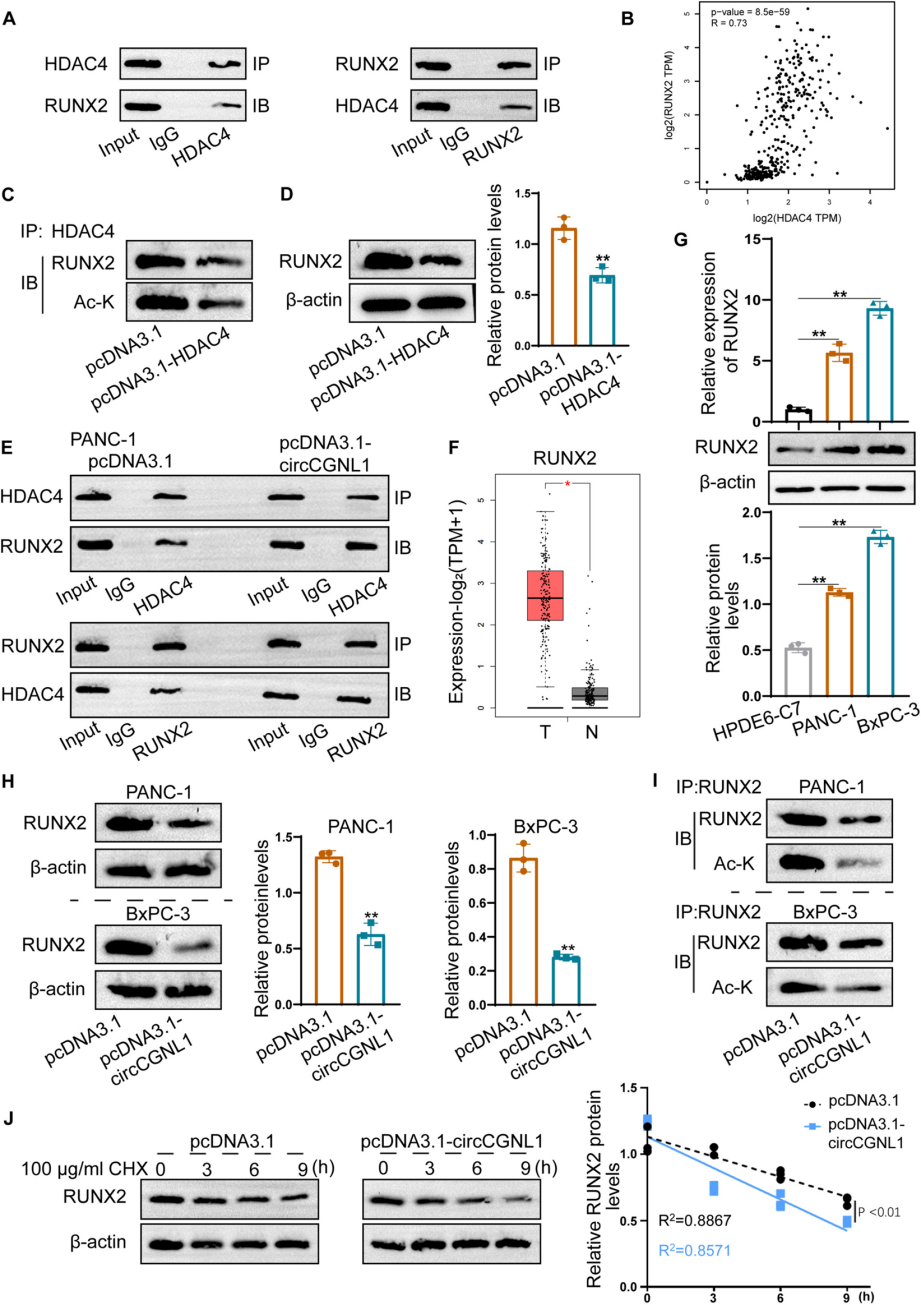
circCGNL1 facilitates HDAC4-induced RUNX2 deacetylation and destabilization. **A** Co-IP assays were performed to detect the interaction between HDAC4 and RUNX2 in 293 T cells. An anti-lgG antibody served as the control. **B** The GEPIA database was used to predict the co-expression relationship between HDAC4 and RUNX2 in PC and normal tissues. A non-log scale was used for calculations, a log-scale axis was used for visualization, and Spearman’s correlation coefficient was determined. **C**, **D** Acetylation and protein expression levels of RUNX2 in HDAC4-overexpressing 293 T cells were estimated using IP-WB assays. **E** Co-IP assays were performed to analyze the influence of circCGNL1 overexpression on the interplay between RUNX2 and HDAC4. **F** The GEPIA database was used to assess RUNX2-expression levels in PC tissues and normal tissues. RUNX2 expression level was defined by log2(TPM + 1), number of pancreatic tumor tissues (T) = 179, number of normal pancreatic tissues (N) = 171. **G** The mRNA and protein expression levels of RUNX2 in PANC-1 and BxPC-3 cells were detected using qRT-PCR and WB analyses. **H** WB assays were performed to detect the protein expression levels of RUNX2 in PANC-1 and BxPC-3 cells transfected with pcDNA3.1-circCGNL1. **I** Co-IP-WB assays were performed to study the influence of circCGNL1 overexpression on RUNX2 acetylation. **J** PANC-1 cells were transfected with a circCGNL1-overexpression plasmid or the empty vector and treated with Cycloheximide (CHX) at 100 μg/ml. WB analysis was performed to detect RUNX2 stability at 3, 6, and 9 h (h) after CHX treatment (HY-12320, MCE, USA). Relative RUNX2 protein level was normalized with β-actin. R^2^: Goodness of fit, *p < 0.05, **p < 0.01

Nuclear HDAC4 can drive RUNX2 deacetylation and degradation [30]. We demonstrated that circCGNL1 promotes dephosphorylation and nuclear transfer of HDAC4 via NUDT4. Therefore, we further studied whether circCGNL1 regulates RUNX2 deacetylation and degradation in an HDAC4-mediated manner. First, we verified the regulatory effect of HDAC4 on RUNX2 acetylation. Co-IP assays showed that HDAC4 interacted with RUNX2 (Fig. 6A), which was supported by GEPIA analysis results (Fig. 6B). Furthermore, pcDNA3.1-HDAC4 transfectants (Supplementary Fig. 1I) revealed that HDAC4 upregulation clearly reduced RUNX2 protein acetylation and expression in 293 T cells (Fig. 6C,D). Co-IP assays showed that circCGNL1 overexpression promoted the HDAC4–RUNX2 interaction in PANC-1 cells (Fig. 6E). In addition, GEPIA data showed that RUNX2 expression was notably higher in PC tissues than in normal control tissues (Fig. 6F). qRT-PCR and WB assays showed that RUNX2 was also expressed at higher levels in PANC-1 and BxPC-3 cells (Fig. 6G). circCGNL1 upregulation inhibited both RUNX2 expression and acetylation in PANC-1 and BxPC-3 cells (Fig. 6H,I), facilitating RUNX2 degradation under CHX treatment (Fig. 6J). In summary, circCGNL1 enhanced HDAC4-mediated RUNX2 deacetylation to promote RUNX2 protein degradation.

### RUNX2 transcriptionally inhibits GAMT expression

**Fig. 7:**
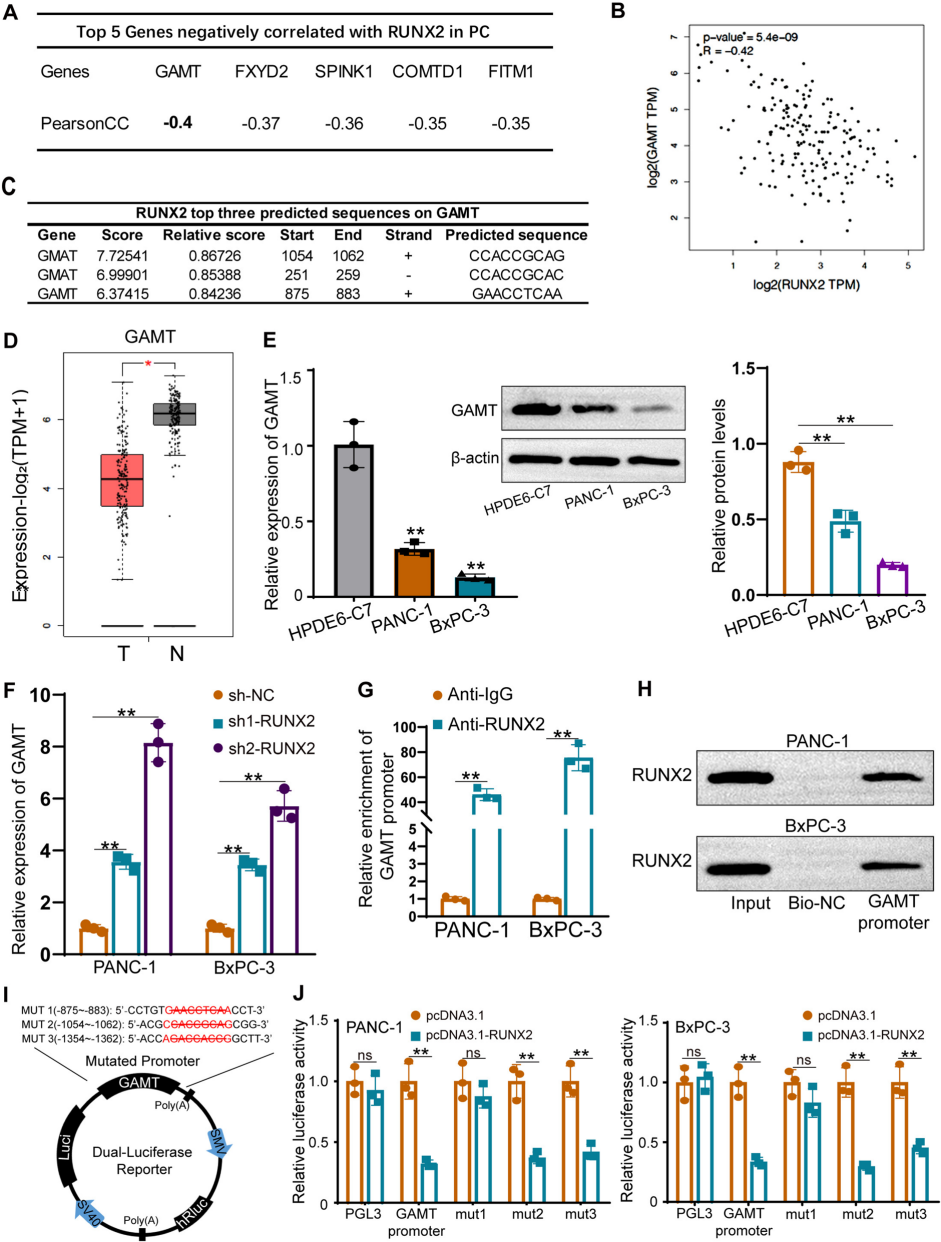
RUNX2 transcriptionally inhibits GAMT expression. **A** The online UALCAN tool was used to predict genes that correlate negatively with RUNX2 expression in PC cells, based on TCGA data. Candidate downstream genes and Pearson correlation coefficients (PearsonCC) are shown. **B** The GEPIA database was used to predict GAMT and RUNX2 co-expression in PC cells. A non-log scale was used for calculations, a log-scale axis was used for visualization, and Spearman’s correlation coefficient was determined. **C** JASPAR database analysis revealed potential RUNX2-binding sites in the *GAMT* promoter region. The top three predicted binding sites are shown. **D** GAMT expression in PC tissues and normal tissues was studied using the GEPIA database. GAMT expression level was defined by log2(TPM + 1), number of pancreatic tumor tissues (T) = 179, number of normal pancreatic tissues (N) = 171. **E** GAMT mRNA and protein expression levels in PANC-1 and BxPC-3 cells were detected using qRT-PCR and WB analysis, respectively. **F** qRT-PCR analysis of GAMT expression in BxPC-3 and PANC-1 cells when RUNX2 was silenced. **G**, **H** ChIP and DNA pulldown assays were used to verify the interaction between RUNX2 and the *GAMT* promoter. **I** Schematic diagram of the luciferase reporter plasmids with three mutated *GAMT* promoter sequences (MUT1-MUT3). **J** WT and mutated *GAMT* promoters were used in dual-luciferase reporter assays. ns: no significance, **p < 0.01

As RUNX2 is a common transcription factor, we further explored the target genes downstream of RUNX2 in PC cells. UALCAN database (http://ualcan.path.uab.edu/) was used to predict the genes related to RUNX2 in PC. GAMT ranked first among the genes negatively correlated with RUNX2 in PC (Fig. 7A). GEPIA database also showed a negative correlation between GAMT and RUNX2 expression in PC tissues (Fig. 7B). The JASPAR database (http://jaspar.genereg.net/) revealed potential RUNX2-binding sites on the *GAMT* promoter, and the top three motifs are shown in Fig. 7C. The GEPIA database was used to analyze GAMT expression in 179 PC tissues and 171 normal tissues; GAMT was significantly downregulated in PC tissues (Fig. 7D). qRT-PCR and WB confirmed that GAMT expression was lower in PANC-1 and BxPC-3 cells (Fig. 7E). We knocked down RUNX2 in PANC-1 and BxPC-3 cells, and qRT-PCR and WB were performed to verify transfection efficiency (Supplementary Fig. 1J). RUNX2 knockdown considerably elevated GAMT expression (Fig. 7F), which was consistent with our bioinformatic analysis results. ChIP and DNA pulldown assays showed that RUNX2 bound the *GAMT* promoter (Fig. 7G,H). Next, we overexpressed RUNX2 in PANC-1 and BxPC-3 cells (Supplementary Fig. 1K) transfected with luciferase reporter plasmids encoding either WT or mutant *GAMT* promoters (Fig. 7I). Ectopic RUNX2 expression significantly reduced the luciferase activity of the WT *GAMT* promoter and two *GAMT* promoter mutants (mut2 and mut3) but did not evidently influence that of the mut1 *GAMT* promoter (Fig. 7J). These data showed that RUNX2, acted as a transcription factor, could bind to site 1 (-875 to -883) of the *GAMT* promoter to hinder GAMT transcription.

### RUNX2 promotes PC cell progression by suppressing GAMT-mediated apoptosis

**Fig. 8:**
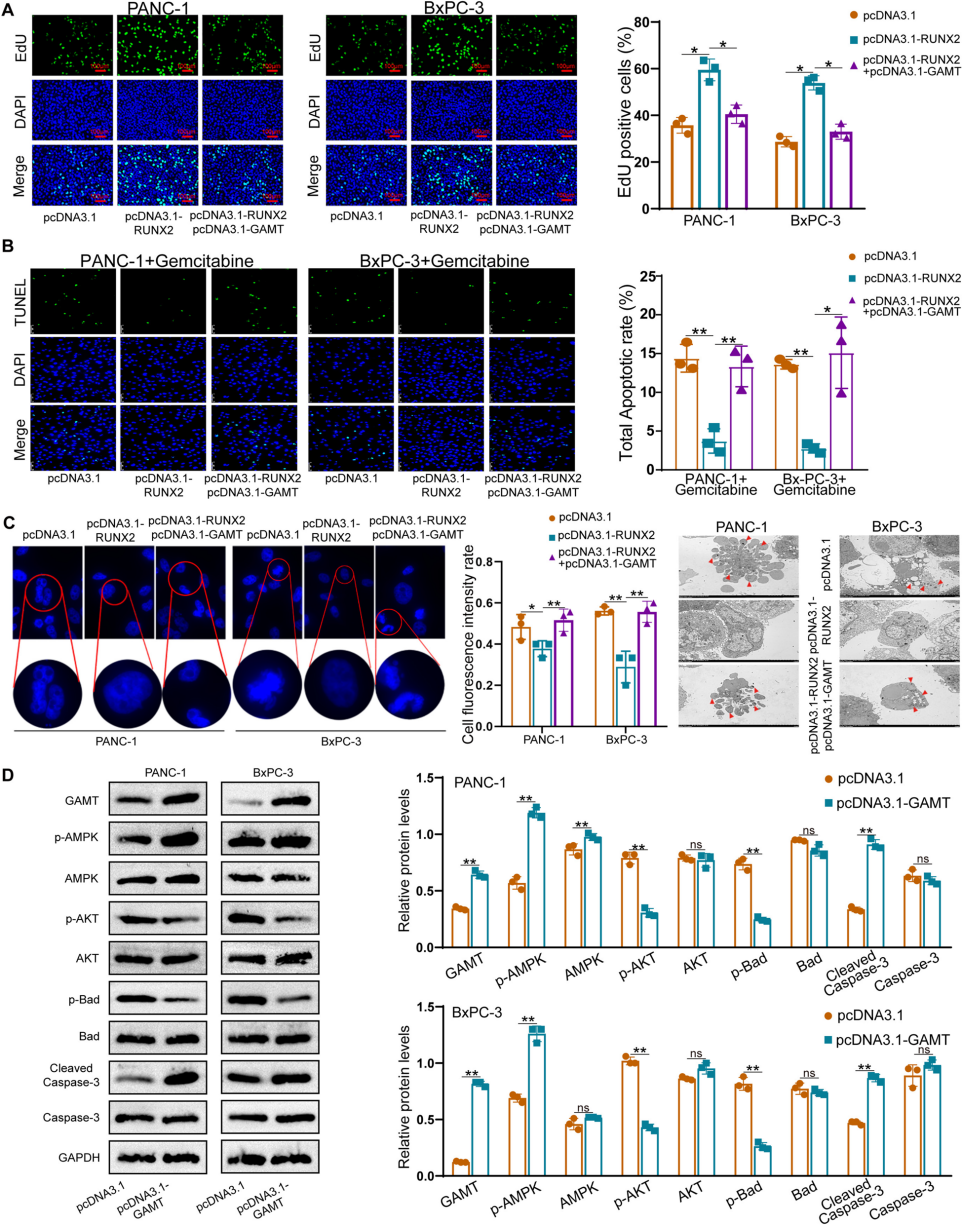
RUNX2 promotes PC progression by repressing GAMT. **A** Proliferation of PANC-1 and BxPC-3 cells transected with an RUNX2-overexpression vector, co-transfected with RUNX2-GAMT overexpression vectors, or the pcDNA3.1 vector control was studied using EdU assays. **B** TUNEL assays were used to detect PC cells apoptotic rate. Gemcitabine (HY-17026, MCE) was used to treat PANC-1 and BxPC-3 cells at 1 μmol/L for 24 h to induce cell apoptosis. **C** DAPI staining assay were applied to detect PC cell apoptosis. Cell fluorescence intensity was measured by ImageJ. PC cell nuclear chromatin morphological changes were observed by TEM. The red arrows showed the apoptotic bodies. **D** Expression levels of proteins related to apoptosis. Protein extracts from pcDNA3.1-GAMT and pcDNA3.1 transfectants analyzed using WB assay to detect total and phosphorylated AMPK, AKT, Bad, and cleaved caspase-3. ns: no significance, *p < 0.05, **p < 0.01

We then performed function-rescue assays to verify the regulatory role of the RUNX2–GAMT pathway in PC progression. GAMT upregulation was confirmed in two types of PC cell pcDNA3.1-GAMT transfectants (Supplementary Fig. 1L). EdU and CCK-8 assays showed that PC cell proliferation was promoted in pcDNA3.1-RUNX2 transfectants, and this effect was offset by pcDNA3.1-GAMT co-transfection (Fig. 8A and Supplementary Fig. 6B,C). TUNEL and Flow cytometric assays demonstrated that RUNX2 could repress PC cell apoptosis, which was prevented by co-overexpressing GAMT (Supplementary Fig. 6D-G). In addition, gemcitabine was used to treat PANC-1 and BxPC-3 cells to induce cell apoptosis. CCK-8 and TUNEL assays were further performed to detect PC cell viability and apoptosis after gemcitabine treatment, which also showed GAMT could rescued the impact of RUNX2 (Fig. 8B and Supplementary Fig. 7A). These results were consistent with the DAPI staining assay and nucleus shrinks could be observed by TEM (Fig. 8C). Having validated the effect of GAMT on PC cell apoptosis, we attempted to unveil the underlying mechanism. Caspase-3 is a terminal splicing enzyme that plays an essential role in cell apoptosis. Hence, we detected the levels of several well-characterized pro-apoptotic signaling molecules using WB. GAMT overexpression decreased the levels of phosphorylated AKT and Bad but increased those of phosphorylated AMPK and cleaved caspase-3 relative to those in control cells (Fig. 8D). We also knocked down AMPK in BxPC-3 and PANC-1 cells by AMPK-siRNAs (Supplementary Fig. 7B). AMPK-siRNA3 was used to perform functional experiment. TUNEL assay demonstrated circCGNL1 induced PANC-1 and BxPC-3 cells apoptosis, however, the function could be attenuated by knocking down AMPK (Supplementary Fig. 7C,D). These results indicated that RUNX2 promoted PC progression by suppressing the pro-apoptotic AMPK–AKT–Bad–cleaved caspase-3 signaling pathway in PC cells in a GAMT-dependent manner, as indicated schematically in Supplementary Fig. 7E.

### CircCGNL1 represses PC tumor growth in vivo

**Fig. 9:**
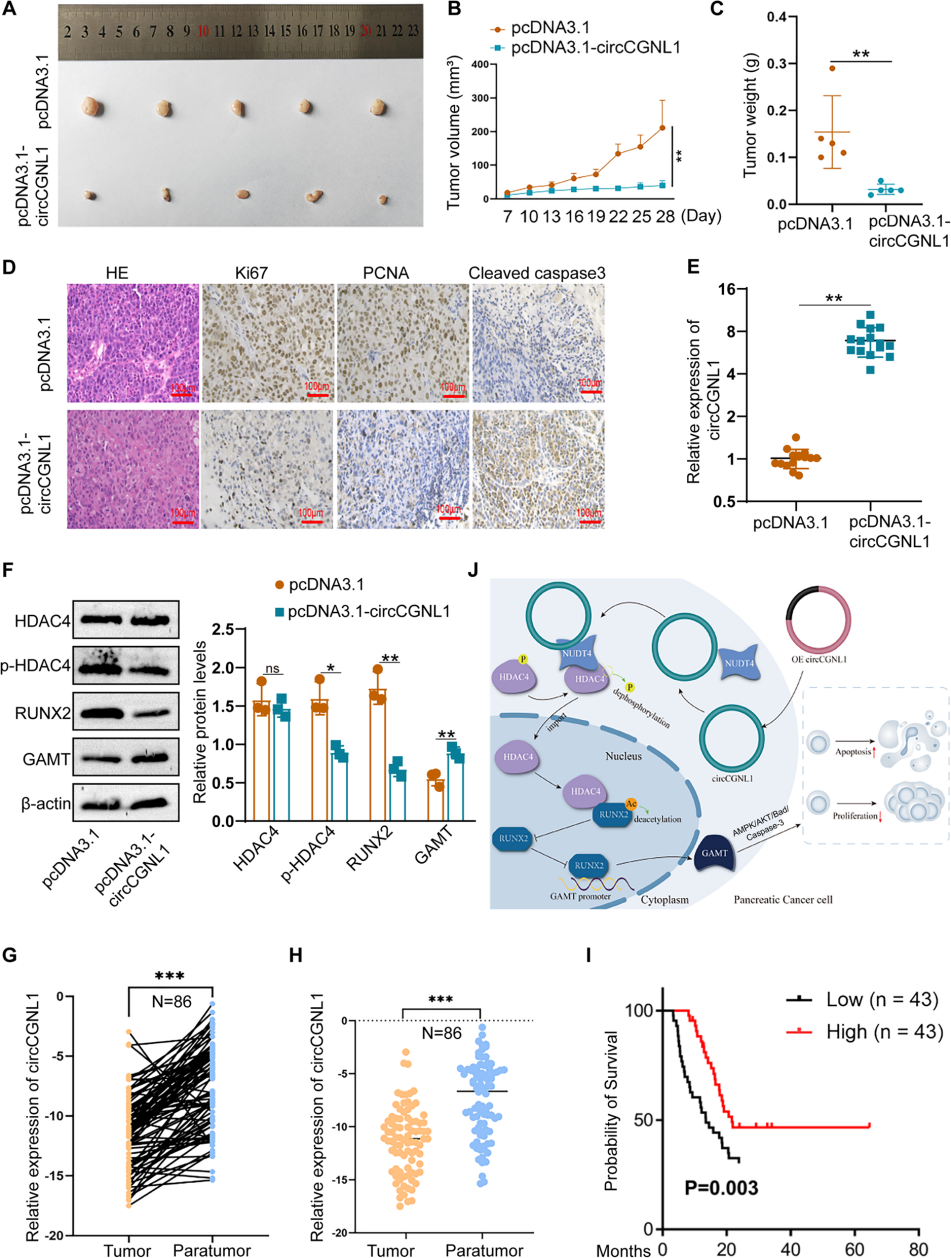
circCGNL1 represses PC tumor growth in vivo. **A** Representative images of subcutaneous xenograft tumors (*n* = 5/group), showing that circCGNL1 suppressed the tumorigenesis of PANC-1 cells in vivo. **B**, **C** Analysis of tumor volumes and weights revealed decreased tumor growth after ectopic expression of circCGNL1 in PANC-1 cells. **D** Hematoxylin and eosin (HE) and IHC staining of xenograft tumors. Protein expression levels of Ki67, PCNA, and cleaved caspase-3 were analyzed using IHC staining. The samples were imaged at 100 × magnification (Scale bar = 100 μm). **E** qRT-PCR was performed to detect circCGNL1 expression in tumor tissues from the negative control and pcDNA3.1-circCGNL1 treatment groups. **F** Protein levels of HDAC4, p-HDAC4 (S632), RUNX2, and GAMT were analyzed in circCGNL1-overexpressing and control tumor tissues using WB assays. **G**, **H** Relative circCGNL1-expression levels in PC tissues (tumor) and adjacent non-tumor tissues (paratumor) via RT-qPCR (*n* = 86). The samples were collected from The First Affiliated Hospital of Nanjing Medical University were detected. **I** Kaplan–Meier survival curves revealing the OS of PC patients with low versus high circCGNL1 expression. The median expression level of circCGNL1 was set as the cut-off value. **J** Schematic illustration indicates the mechanism of circCGNL1 in regulating PC growth via NUDT4–HDAC4–RUNX2–GAMT-mediated apoptosis. ns: no significance, **p < 0.01

Finally, we detected the effect of circCGNL1 on PC tumor growth in vivo. PANC-1 cells were transfected with either the pcDNA3.1-circCGNL1 or empty pcDNA3.1 vector, after which the cells were subcutaneously injected into BALB/c mice. Tumor-bearing mice were euthanized at 28 days after breeding (Supplementary Fig. 7F). The tumors derived from circCGNL1-overexpressed cells exhibited significantly lower size (average volume, 211mm^3^ vs 40mm^3^) and weight (average weight, 154 mg vs 32 mg) than those from the control group (Fig. 9A–C). Furthermore, IHC showed that tumors with circCGNL1 overexpression expressed less Ki67 (MKI67, marker of proliferation Ki-67) and PCNA (Proliferating Cell Nuclear Antigen) and more cleaved caspase-3 than the control tumors (Fig. 9D). qRT-PCR demonstrated that circCGNL1 expression was higher in tumors originating from pcDNA3.1-circCGNL1 transfectants cells (Fig. 9E). WB of the harvested tumor tissues showed that circCGNL1 overexpression significantly inhibited HDAC4 phosphorylation and RUNX2 expression but promoted GAMT expression (Fig. 9F). Eighty-six PC patient samples were collected from the First Affiliated Hospital of Nanjing Medical University for further examination. qRT-PCR results revealed lower circCGNL1 expression in PC samples than in adjacent normal tissues (Fig. 9G-H). These results were consistent with separate patient samples results obtained from Heping Hospital Affiliated of Changzhi Medical College. The relationship between circCGNL1 expression and the clinical characteristics of the PC patients from Nanjing and Changzhi cohorts are listed in Table 1. Microscopic vascular and nerve invasion were both significantly associated with circCGNL1 in the two cohorts. The tumor size and grade were statistically correlated with circCGNL1 in Changzhi cohort. Further univariate and multivariate Cox regression analysis indicated that circCGNL1 and serum CA199 level were independent prognostic factors for PC patients in both cohorts (Table 2). In addition, patients with lower circCGNL1 expression had a poorer OS than patients with higher circCGNL1 expression (Fig. 9I). Therefore, circCGNL1 can hinder PC tumor growth in vivo.

**Table 1:**
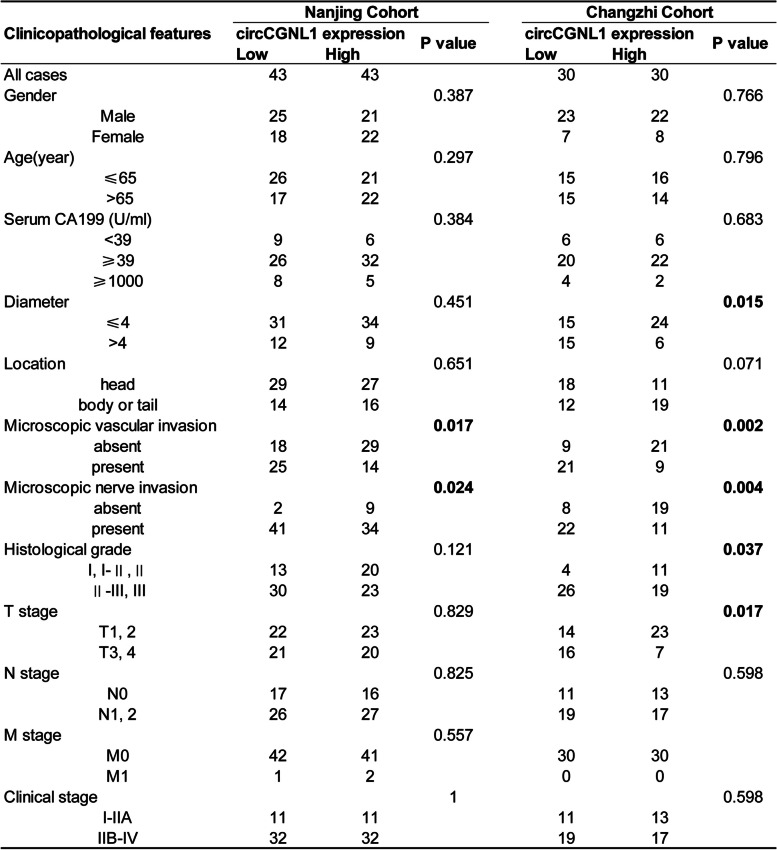
Relationship between circCGNL1 expression and PC patients clinicopathological features (*n* = 146)

**Table 2:**
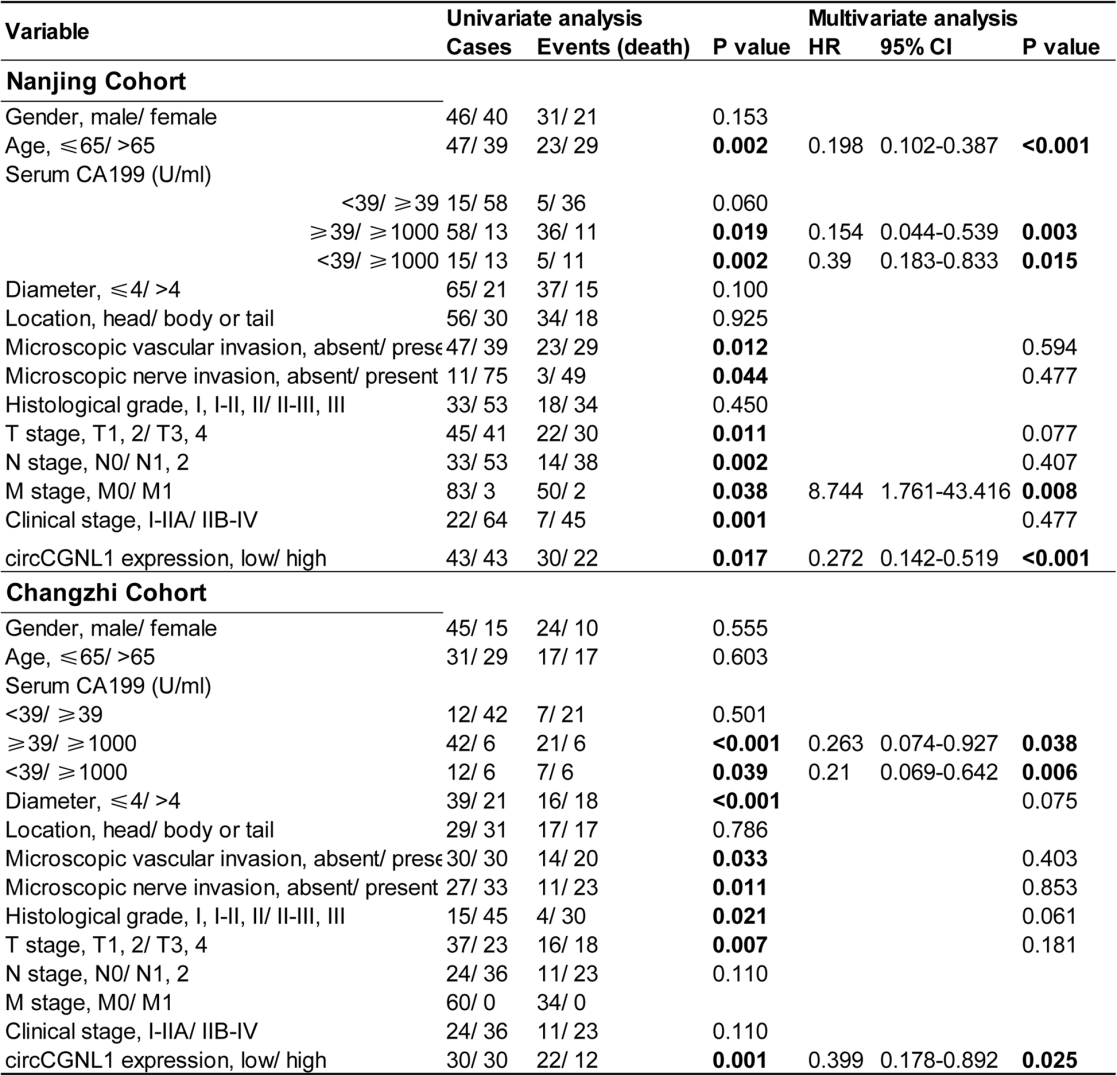
Univariate and multivariate analyses of prognostic factors in PC patients (*n* = 146)

## Discussion

PC is a highly malignant tumor of the digestive tract that is extremely difficult to diagnose and treat [31]. CircRNAs play essential roles in tumorigenesis and cancer progression, and their high degree of sequence conservation and relative stability are crucial for their properties [32]. CircRNA dysregulation has been identified in assorted cancers, but the associated roles and mechanisms remain unclear in most cases [33, 34]. We aimed to determine the biological function and regulatory mechanism of circCGNL1 in PC. Here, we discovered a novel circRNA, circCGNL1, that was expressed at low levels in PC tissues and cells. Therefore, we suspected that circCGNL1 acts as a tumor suppressor in PC. We performed gain and loss-of-function assays to determine its biological functions in vitro and in vivo. We found that circCGNL1 overexpression repressed cell proliferation and promoted PC cell apoptosis in vitro, and inhibited tumor growth in vivo. Collectively, these data demonstrated the antitumor properties of circCGNL1 in PC.

It has been reported circRNAs could impact tumor progression vid multiple methods including miRNA Sponge, competing endogenous RNAs and RNA Binding Protein [9]. To investigate the regulatory mechanism of circCGNL1 in PC, we performed RNA pulldown assays and bioinformatic analysis to identify the NUDT4 as a downstream target that could bind with circCGNL1. However, circCGNL1 did not affect NUDT4 expression. As a phosphatase, NUDT4 might regulate cellular physiology by its target proteins [35]. Thus, we suspected circCGNL1 recruits NUDT4 to impact its downstream targets. We further predicted NUDT4-interacting proteins using bioinformatic tools and by performing mechanistic assays, revealing HDAC4 could interact with NUDT4.

HDAC4 serves crucial roles in chromatin maintenance and function by modulating histone acetylation [36], its dysregulation has frequently been observed in human cancers. For example, HDAC4 overexpression accelerated epithelial ovarian cancer cell proliferation and migration [37]. HDAC4 also repressed PC apoptosis, and its inhibitors have been evaluated for treating advanced PC [38, 39]. Notably, we found NUDT4 dephosphorylated HDAC4 at Ser632, which is key to the nucleocytoplasmic translocation of HDAC4 [40]. Importantly, our IF-WB assays showed that circCGNL1 increased nuclear HDAC4 and decreased cytoplasmic HDAC4 levels; thus, our results suggest circCGNL1 can promote nuclear HDAC4 translocation by regulating NUDT4.

Histone acetylation plays a crucial role in regulating gene expression in eukaryotes [41, 42]. High acetylation marks active transcription, whereas low acetylation is associated with transcriptional inhibition [43]. circRNAs could participate in cancer progression by modifying histone acetylation [44]. Previous research indicated that reduced CaMKII phosphorylation causes HDAC4 dephosphorylation, which increased HDAC4 distribution in the nucleus, resulting in decreased RUNX2 acetylation [45]. In our study, we discovered that circCGNL1 interacted with NUDT4, leading to HDAC4 dephosphorylation and enhanced translocation of HDAC4 to nucleus. Therefore, we further investigated the role of RUNX2 acetylation in PC. Co-IP and IP-WB assays confirmed that circCGNL1 inhibited RUNX2 acetylation and promoted RUNX2 degradation. RUNX2 is a member of the RUNX family of metazoan transcription factors, which are important regulators of cell proliferation, differentiation, and apoptosis in multiple cancers [46]. Hence, we explored the target genes of RUNX2 via bioinformatic tools. GAMT and RUNX2 were found to be negatively correlated in PC tissues, and GAMT expression level was significantly lower in PC. A series of experiments showed that RUNX2 bound the GAMT promoter and inhibited GAMT expression. GAMT overexpression could also reverse the effects of RUNX2 on PC proliferation and apoptosis. Additionally, we unveiled the signaling pathway by which GAMT induced PC cell apoptosis. Currently, studies on regulation of GAMT in cancer are still scarce. Our research also has some limitations. For instance, the upstream mechanism of circCGNL1 and the relationship between circCGNL1 and tumor microenvironment weren’t explored, which will be important subjects of future research.

In conclusion, we unveiled the circCGNL1–NUDT4–HDAC4–RUNX2–GAMT axis formed an apoptosis-activation signaling pathway that regulated PC cell proliferation (Fig. 9J). Our findings provide new insights that may help discern the molecular mechanism underlying PC and discover new biomarkers for PC prognosis.

### Supplementary Information

**Additional file 1. **

**Additional file 2:** **Supplementary Fig. 1.** Transfection efficiency and functions of plasmids and shRNAs in vitro. A, B Subcellular-fractionation and FISH assays to determine subcellular localization of circCGNL1 in MIA-PaCa-2 and Capan-1 cells. ACTB (cytoplasm) and U6 (nucleus) were served as internal controls. C qRT-PCR was performed to determine the overexpression efficiency of circCGNL1 in PANC-1 and BxPC-3 cells. D-F Flow cytometric assays were conducted to measure the influence of circCGNL1 overexpression on apoptosis rate. G, H TUNEL and CCK8 assays were performed to detect cells apoptosis (G) and viability (H) in MIA-PaCa-2 and Capan-1 cell lines after knocking down circCGNL1. I qRT-PCR and WB assays showing the overexpression efficiency of HDAC4 plasmid in PANC-1 and BxPC-3 cells. J, K RUNX2 shRNAs (J) and overexpression plasmids (K) were transfected into PANC-1 and BxPC-3 cells, and RUNX2 expression levels were measured using qRT-PCR and WB. L The pcDNA3.1-GAMT plasmid was used to generate GAMT-overexpressing PANC-1 and BxPC-3 cell lines. **p < 0.01.

**Additional file 3:** **Supplementary Fig. 2.** The downstream target of circCGNL1. A Anti-Ago2 (Ago2 antibody) and anti-lgG (IgG antibody) were used to perform RIP experiment to enrich circCGNL1 in PANC-1 cells. B The heatmap shows 180 differentially expressed proteins between PC and pancreatic control tissues based on information in TCGA and GTEx databases. N: Normal pancreatic tissues, T: Pancreatic cancer tissues. ns: no significance.

**Additional file 4:** **Supplementary Fig. 3.** Expression levels of potential genes functioning downstream of circCGNL1. A Volcano plots showing 70 upregulated and 110 downregulated candidate downstream genes based on TCGA and GTEx databases. B-J Expression levels of nine overlapping candidate genes were detected using the GEPIA database, and the NUDT4 expression trend was consistent with the bioinformatic analysis. The PC tissues are shown in the left column (T), and the control tissues are shown in the right column (N). *p < 0.05.

**Additional file 5:** **Supplementary Fig. 4.** Relationship between NUDT4 and circCGNL1. A The Hum-mPLoc website was used to predict the subcellular location of NUDT4. B, C qRT-PCR and WB assays detected NUDT4 expression when circCGNL1 was overexpressed in PANC-1 and BxPC-3 cells. D The catRAPID website was used to predict potential binding sites between circCGNL1 and NUDT4. ns: no significance.

**Additional file 6:** **Supplementary Fig. 5.** NUDT4 accelerated PC progression. A qRT-PCR was performed to determine the knockdown efficiency of NUDT4 shRNAs in PANC-1 and BxPC-3 cells. B-D CCK-8 and EdU proliferation assays were performed to detect cell viability when NUDT4 was inhibited in BxPC-3 and PANC-1 cells. E, F TUNEL and flow cytometric assays were performed to estimate apoptosis when NUDT4 was inhibited. G, H DAPI staining assay and TEM was used to detect PC cell apoptosis (G) and observe the cell nuclear chromatin morphological changes (H). **p < 0.01.

**Additional file 7:** **Supplementary Fig. 6.** RUNX2 promotes PC progression depending on GAMT. A circCGNL1 was up or down-regulated in BxPC-3 cells and Co-IP assays were performed with NUDT4 or HDAC4 as bait proteins. Immunoblotting showed that the combination of NUDT4 and HDAC4 was increased by circCGNL1. B Western blot assay was used to detect RUNX2 and GAMT expression after RUNX2 transfection or RUNX2-GAMT co-transfection. C CCK-8 assays were performed to detect the viability of PANC-1 and BxPC-3 cells in different transfection groups. D, E PANC-1 and BxPC-3 apoptosis rates were detected using flow cytometry after transfecting RUNX2 or GAMT overexpression plasmid. F, G TUNEL assay was performed to examine cell apoptosis when RUNX2 or GAMT overexpressed. ns: no significance, **p < 0.01.

**Additional file 8:** **Supplementary Fig. 7.** Subcutaneous injections in BALB/c mice. A CCK-8 assays were used to detect PC cells viability in pcDNA3.1 vector group, RUNX2 overexpressing group and RUNX2-GAMT co-overexpressing groups after gemcitabine treatment at 1 μmol/L for 24h. B AMPK siRNAs (si-AMPK-1, si-AMPK-2 and si-AMPK-3) were used to knock down AMPK in BxPC-3 and PANC-1 cells, which was identified via qRT-PCR. C, D TUNEL assay was performed to determine the PC cell apoptosis rate after overexpressing circCGNL1 or knocking down AMPK. E Schematic diagram showing the signaling pathway whereby GAMT mediates PC cell apoptosis. GAMT suppressed AKT phosphorylation by stimulating AMPK phosphorylation and subsequently suppressed Bad phosphorylation and upregulated the downstream effector, cleaved caspase-3, which induced PC cell apoptosis. In contrast, GAMT impairment diminished apoptosis. F BALB/c mice were subcutaneously injected on day 1 with 2 × 10^6^ PANC-1 cells/0.1 ml. Tumor-bearing mice from the circCGNL1-overexpression group and pcDNA3.1 control group were sacrificed at day 28 after breeding, and subcutaneous tumor tissues were harvested and measured. **p < 0.01.

**Additional file 9:** **Supplementary Table 1.** Primers, probes, siRNA and shRNAs used in this study.

**Additional file 10:** **Supplementary Table 2.** The primary and secondary antibodies used in this study.

**Additional file 11:** **Supplementary Table 3.** RNA pulldown assay combined with MS analysis results, showing proteins that have potential to interact with circCGNL1.

**Additional file 12:** **Supplementary Table 4.** Differentially expressed genes (DEGs) between PC and normal control pancreatic tissues, as determined via bioinformatic analysis based on the data from TCGA and GTEx databases.

## Data Availability

All data and materials presented in our study can be found in this published article and supplementary data.
