# Supplementary figures and images for: Role of a novel circRNA-CGNL1 in regulating pancreatic cancer progression via NUDT4–HDAC4–RUNX2–GAMT-mediated apoptosis
Source: Mol Cancer. 2024 Jan 31;23:27. doi: 10.1186/s12943-023-01923-7 (PMC10829403; doi:10.1186/s12943-023-01923-7)

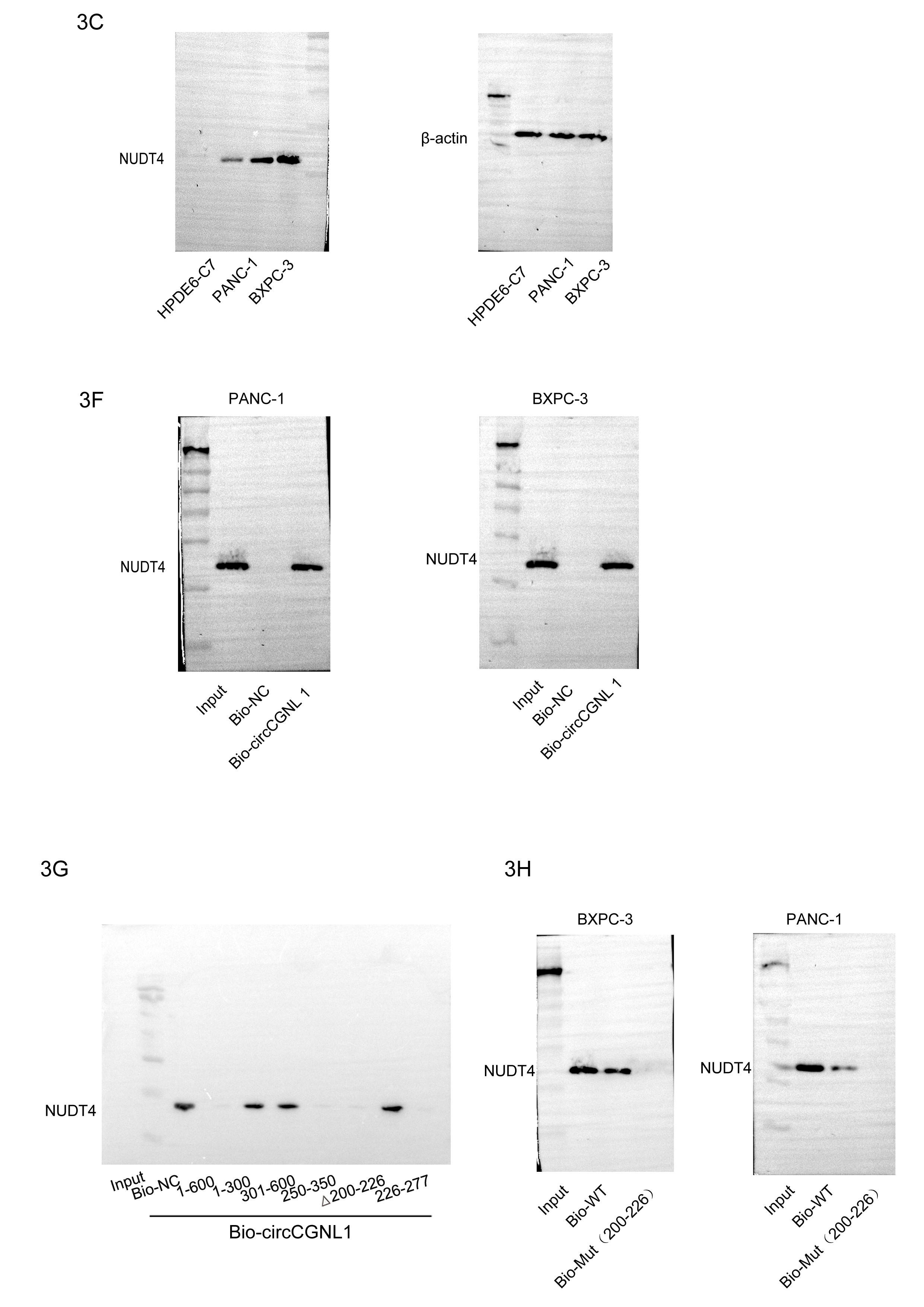

Supplement: Supplementary file 1 — Additional file 1. [file 12943_2023_1923_MOESM1_ESM.zip › Raw Data/Fig 3.tif]

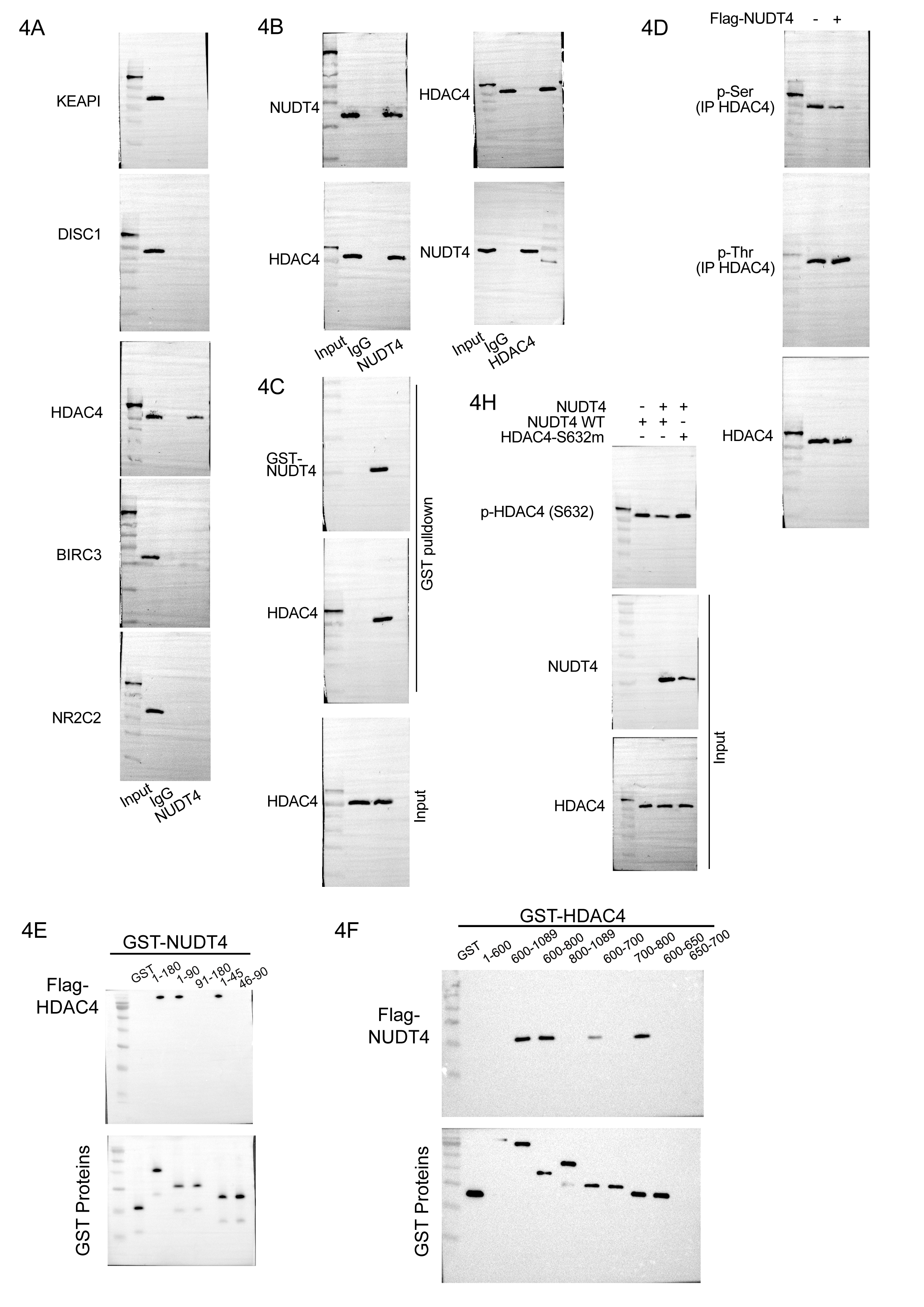

Supplement: Supplementary file 1 — Additional file 1. [file 12943_2023_1923_MOESM1_ESM.zip › Raw Data/Fig 4.tif]

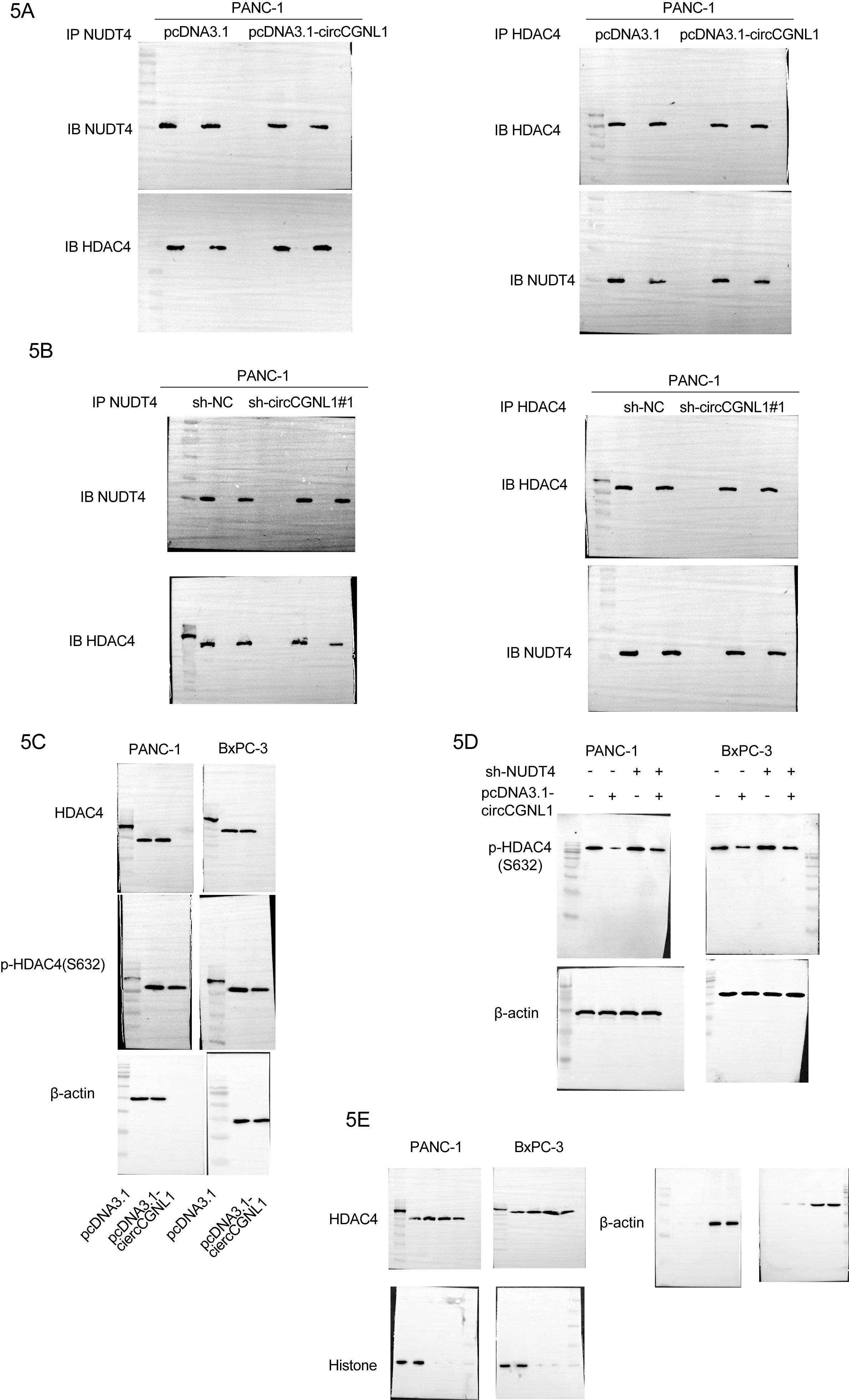

Supplement: Supplementary file 1 — Additional file 1. [file 12943_2023_1923_MOESM1_ESM.zip › Raw Data/Fig 5.tif]

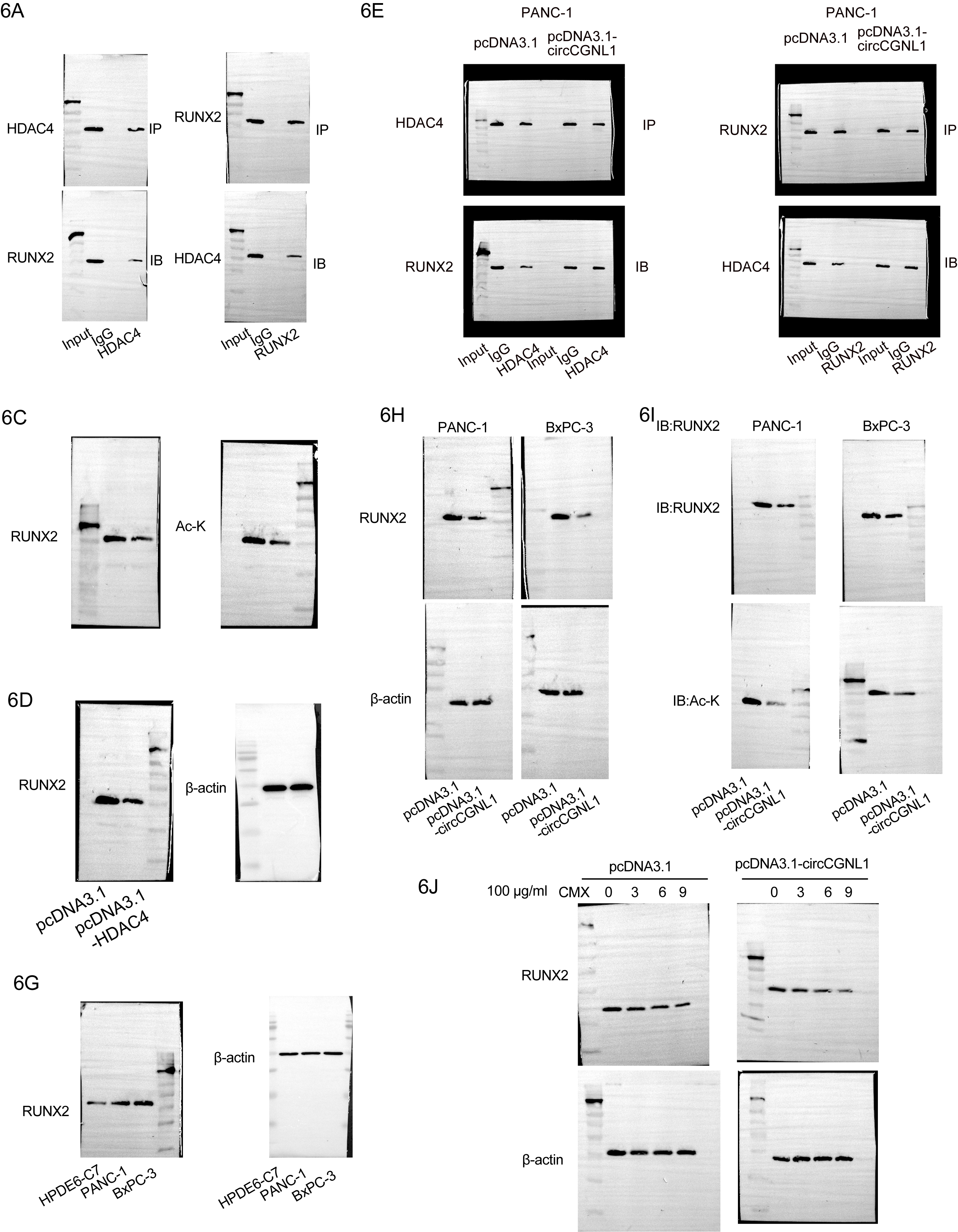

Supplement: Supplementary file 1 — Additional file 1. [file 12943_2023_1923_MOESM1_ESM.zip › Raw Data/fig 6.tif]

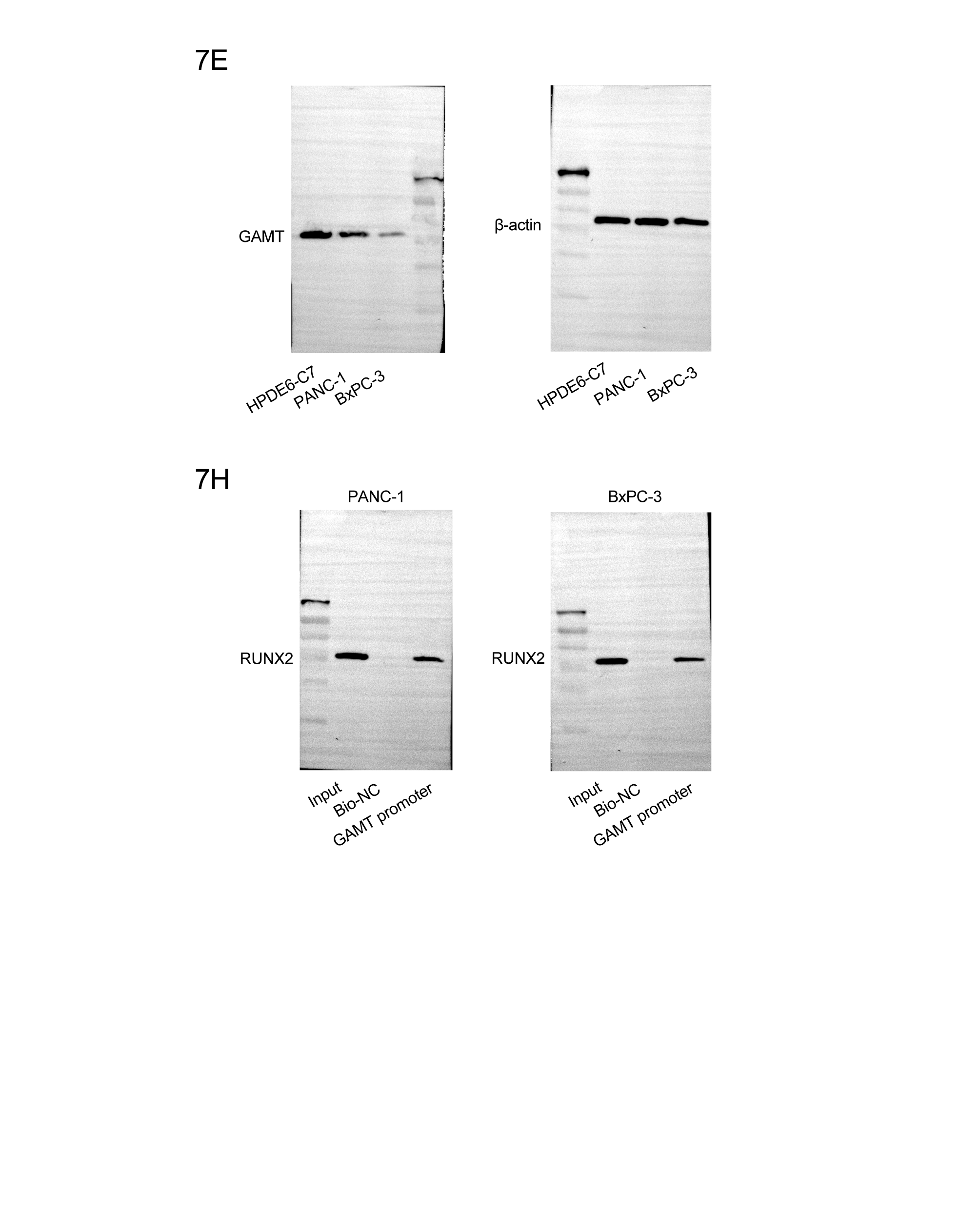

Supplement: Supplementary file 1 — Additional file 1. [file 12943_2023_1923_MOESM1_ESM.zip › Raw Data/Fig 7.tif]

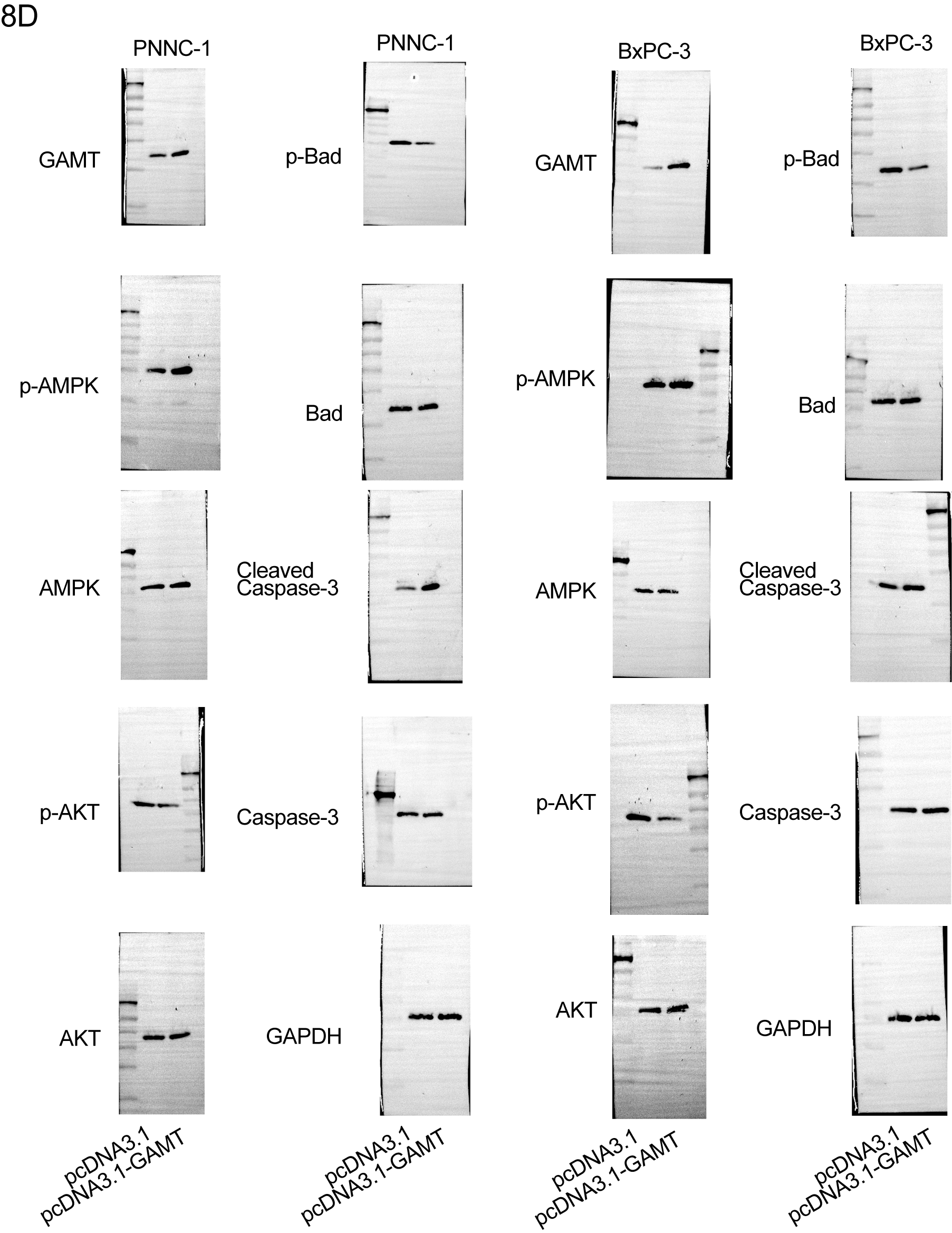

Supplement: Supplementary file 1 — Additional file 1. [file 12943_2023_1923_MOESM1_ESM.zip › Raw Data/Fig 8.tif]

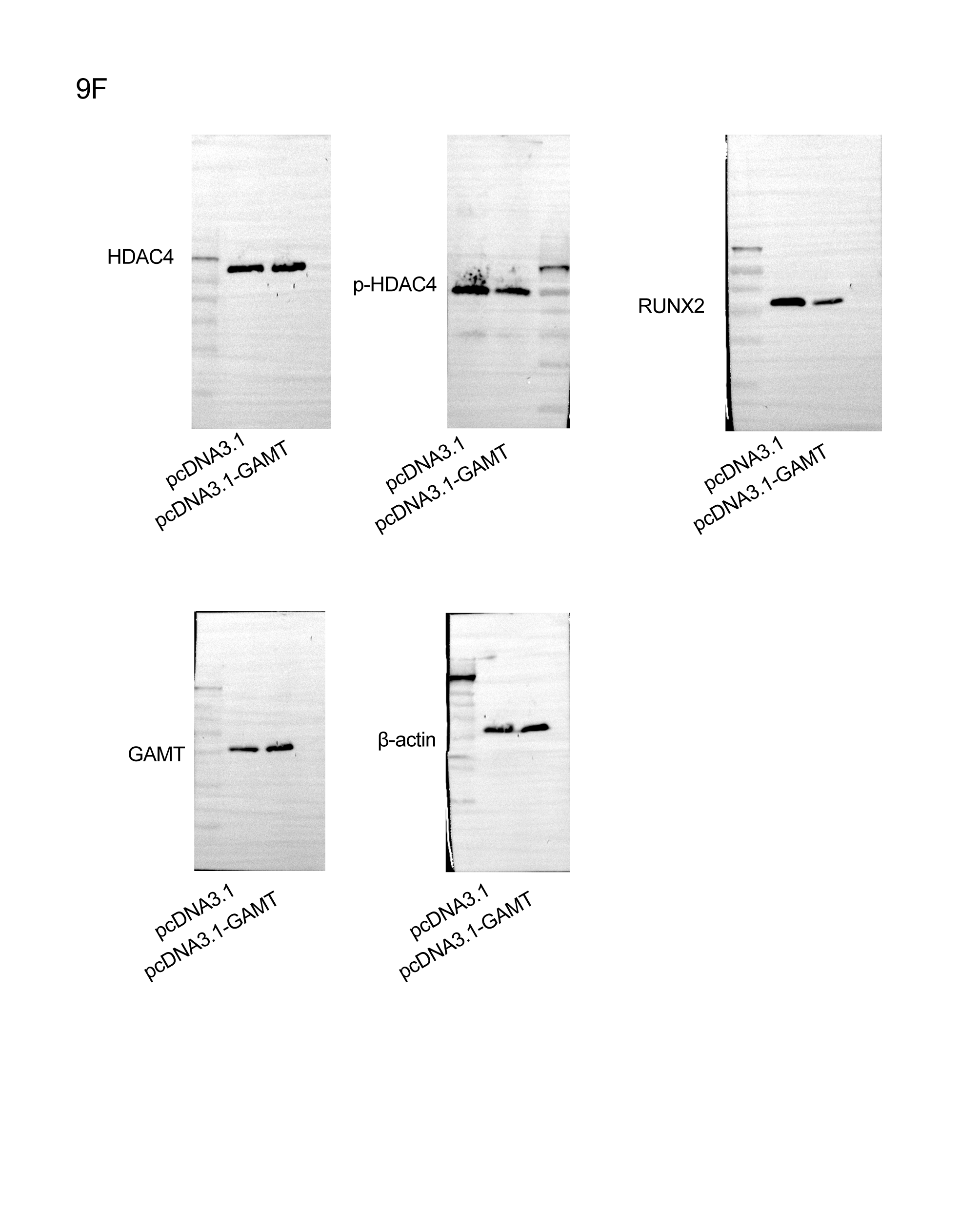

Supplement: Supplementary file 1 — Additional file 1. [file 12943_2023_1923_MOESM1_ESM.zip › Raw Data/Fig 9.tif]

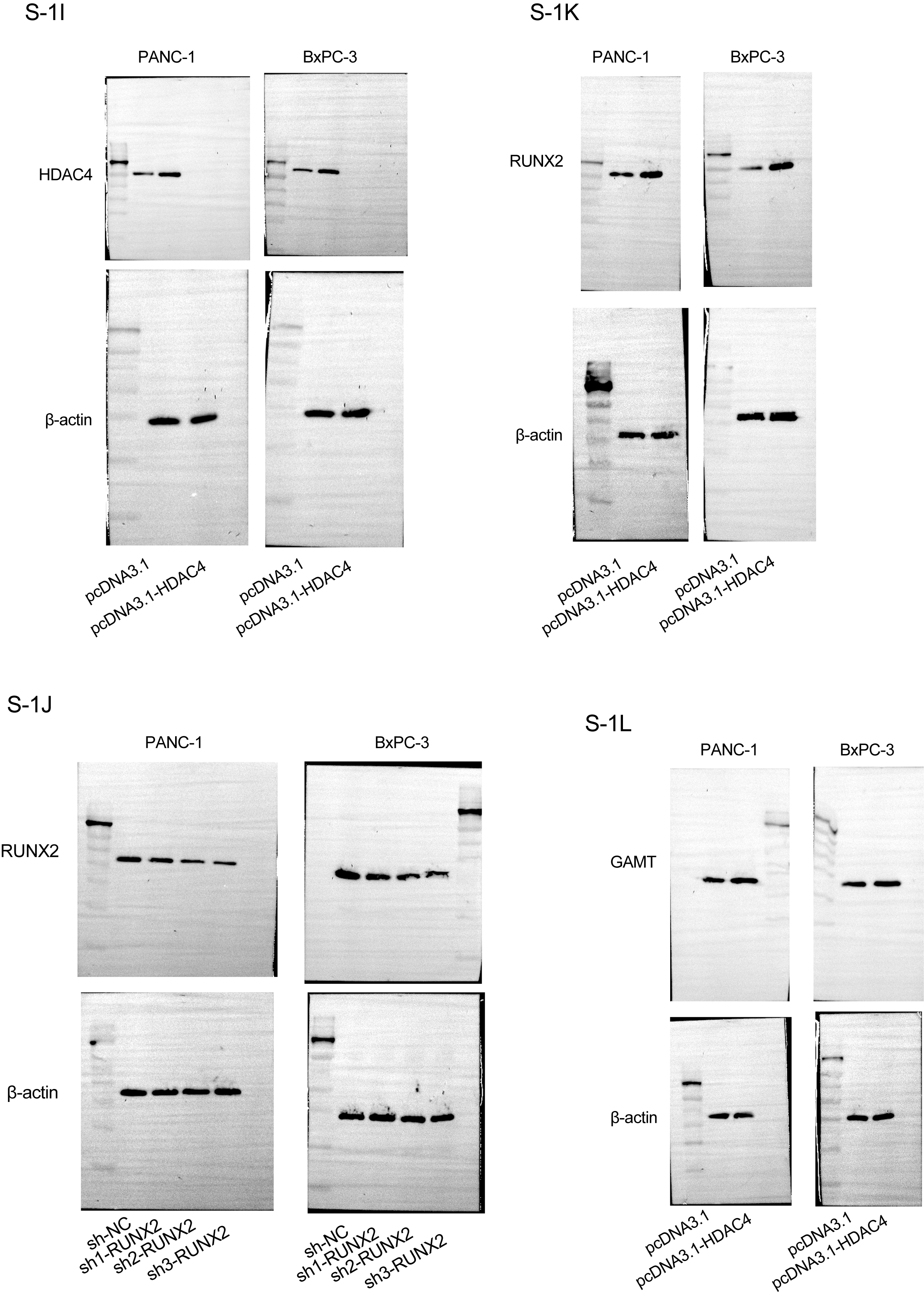

Supplement: Supplementary file 1 — Additional file 1. [file 12943_2023_1923_MOESM1_ESM.zip › Raw Data/Supplementary. Fig 1.tif]

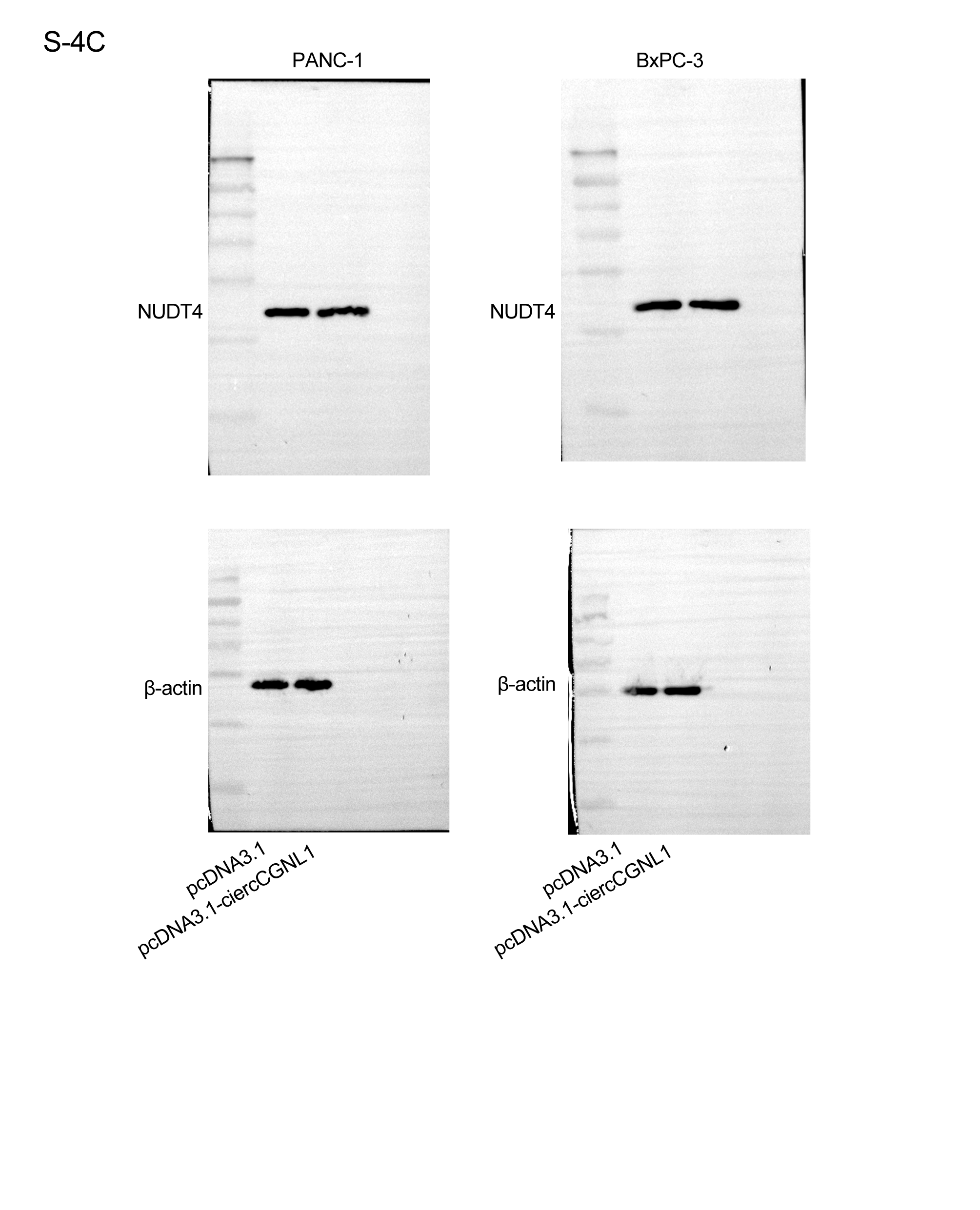

Supplement: Supplementary file 1 — Additional file 1. [file 12943_2023_1923_MOESM1_ESM.zip › Raw Data/Supplementary. Fig 4.tif]

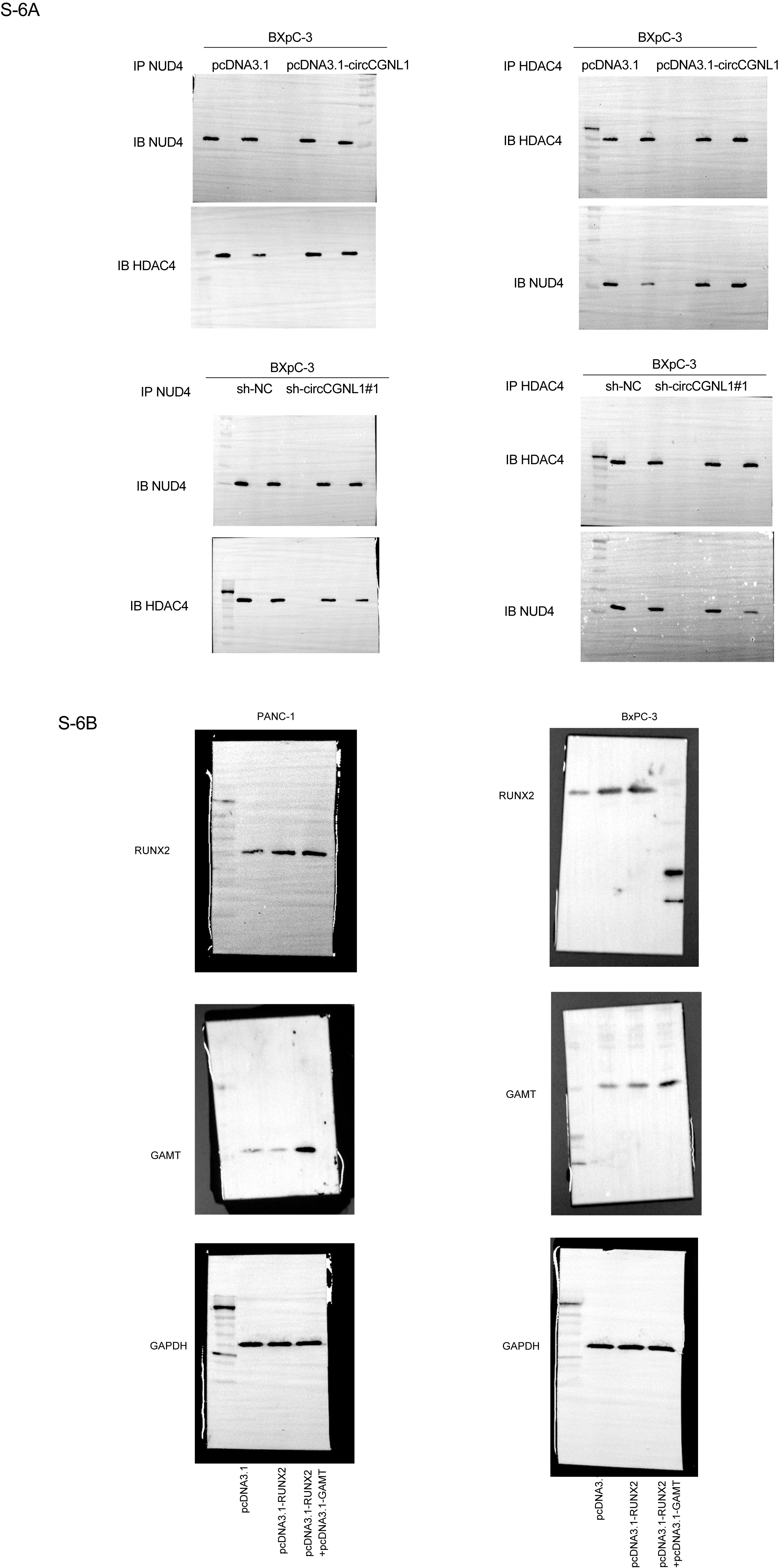

Supplement: Supplementary file 1 — Additional file 1. [file 12943_2023_1923_MOESM1_ESM.zip › Raw Data/Supplementary. Fig 6.tif]

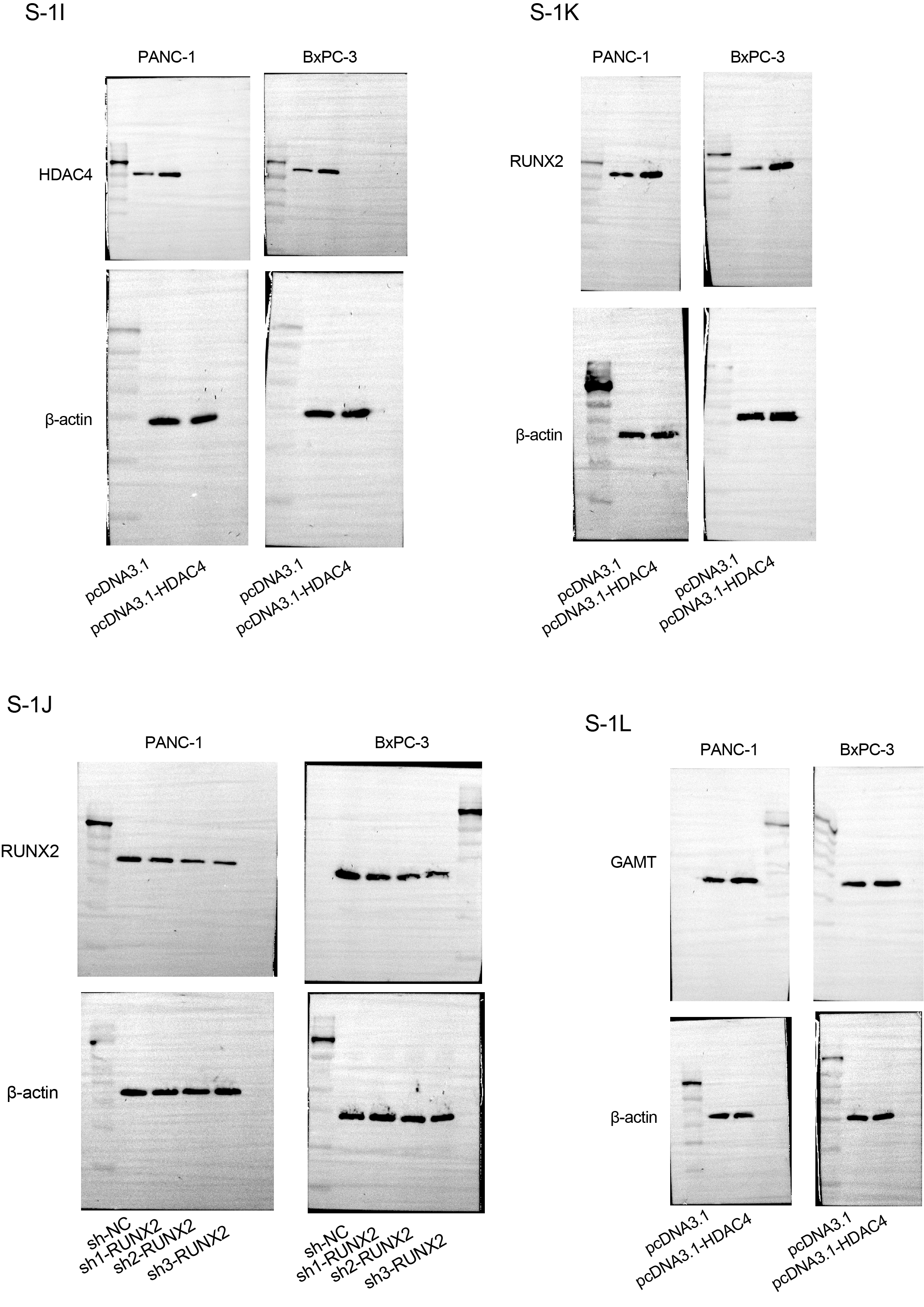

Supplement: Supplementary file 2 — Additional file 2: Supplementary Fig. 1. Transfection efficiency and functions of plasmids and shRNAs in vitro. A, B Subcellular-fractionation and FISH assays to determine subcellular localization of circCGNL1 in MIA-PaCa-2 and Capan-1 cells. ACTB (cytoplasm) and U6 (nucleus) were served as internal controls. C qRT-PCR was performed to determine the overexpression efficiency of circCGNL1 in PANC-1 and BxPC-3 cells. D-F Flow cytometric assays were conducted to measure the influence of circCGNL1 overexpression on apoptosis rate. G, H TUNEL and CCK8 assays were performed to detect cells apoptosis (G) and viability (H) in MIA-PaCa-2 and Capan-1 cell lines after knocking down circCGNL1. I qRT-PCR and WB assays showing the overexpression efficiency of HDAC4 plasmid in PANC-1 and BxPC-3 cells. J, K RUNX2 shRNAs (J) and overexpression plasmids (K) were transfected into PANC-1 and BxPC-3 cells, and RUNX2 expression levels were measured using qRT-PCR and WB. L The pcDNA3.1-GAMT plasmid was used to generate GAMT-overexpressing PANC-1 and BxPC-3 cell lines. **p < 0.01. [file 12943_2023_1923_MOESM2_ESM.jpg]

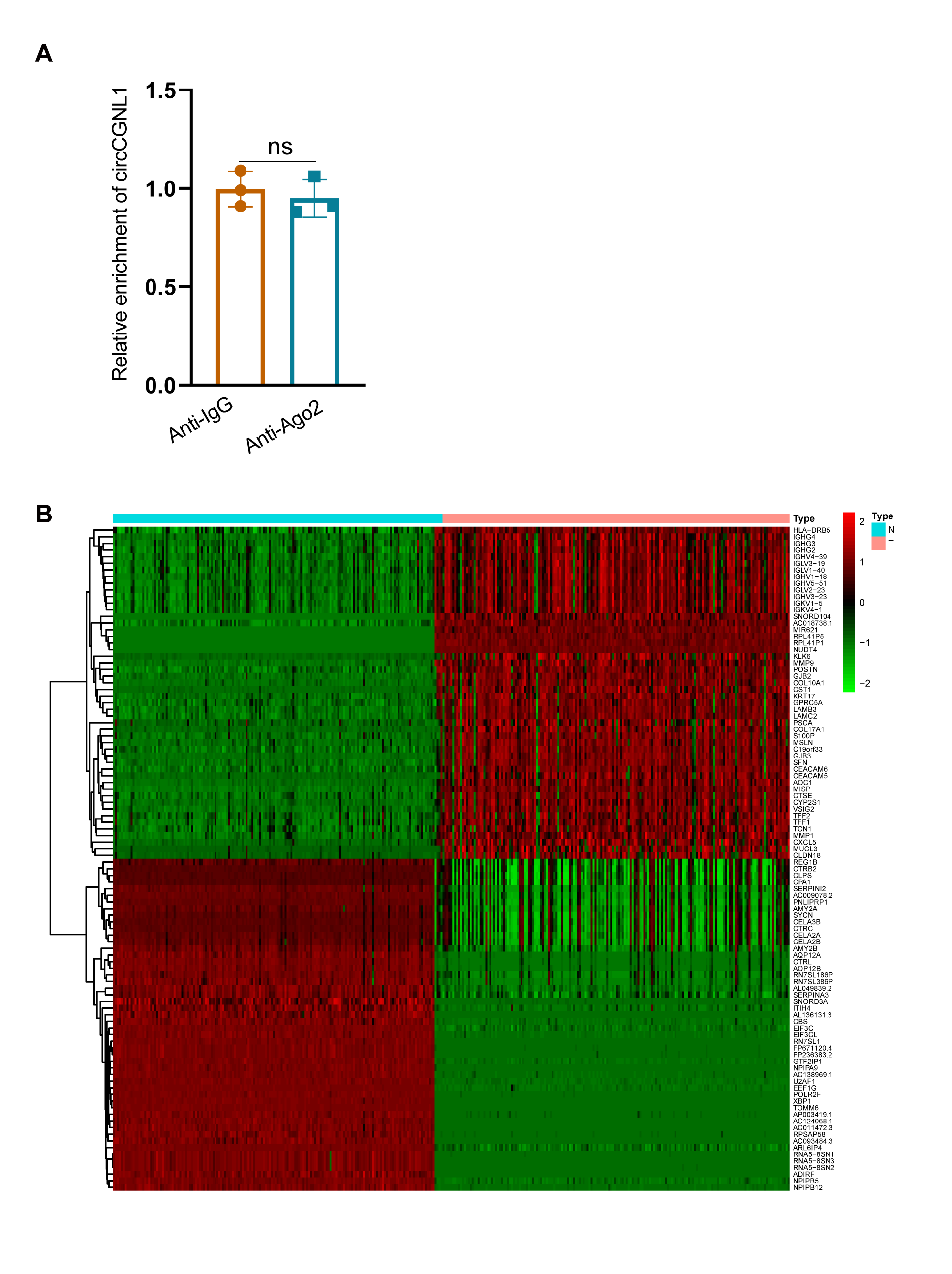

Supplement: Supplementary file 3 — Additional file 3: Supplementary Fig. 2. The downstream target of circCGNL1. A Anti-Ago2 (Ago2 antibody) and anti-lgG (IgG antibody) were used to perform RIP experiment to enrich circCGNL1 in PANC-1 cells. B The heatmap shows 180 differentially expressed proteins between PC and pancreatic control tissues based on information in TCGA and GTEx databases. N: Normal pancreatic tissues, T: Pancreatic cancer tissues. ns: no significance. [file 12943_2023_1923_MOESM3_ESM.tif]

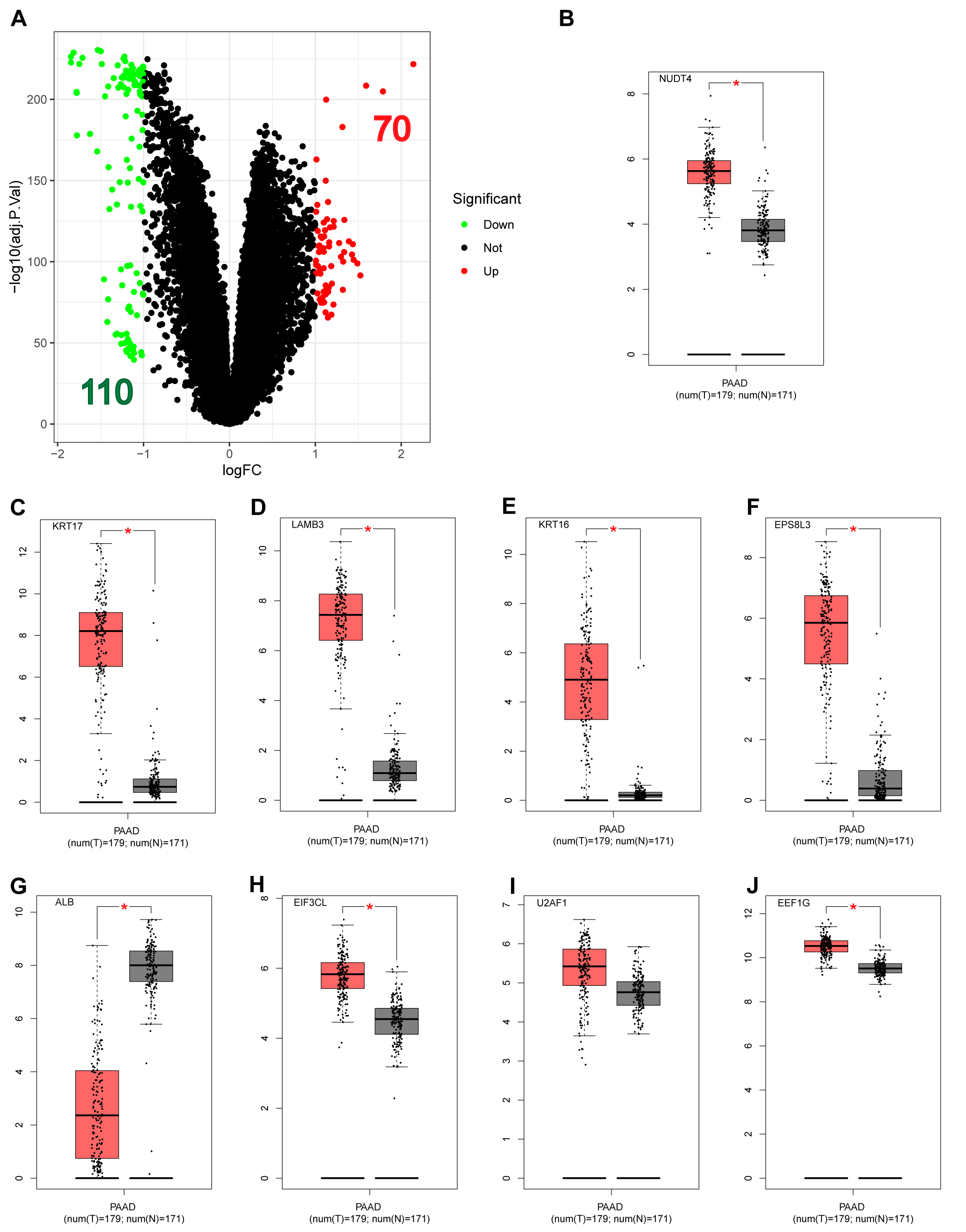

Supplement: Supplementary file 4 — Additional file 4: Supplementary Fig. 3. Expression levels of potential genes functioning downstream of circCGNL1. A Volcano plots showing 70 upregulated and 110 downregulated candidate downstream genes based on TCGA and GTEx databases. B-J Expression levels of nine overlapping candidate genes were detected using the GEPIA database, and the NUDT4 expression trend was consistent with the bioinformatic analysis. The PC tissues are shown in the left column (T), and the control tissues are shown in the right column (N). *p < 0.05. [file 12943_2023_1923_MOESM4_ESM.tif]

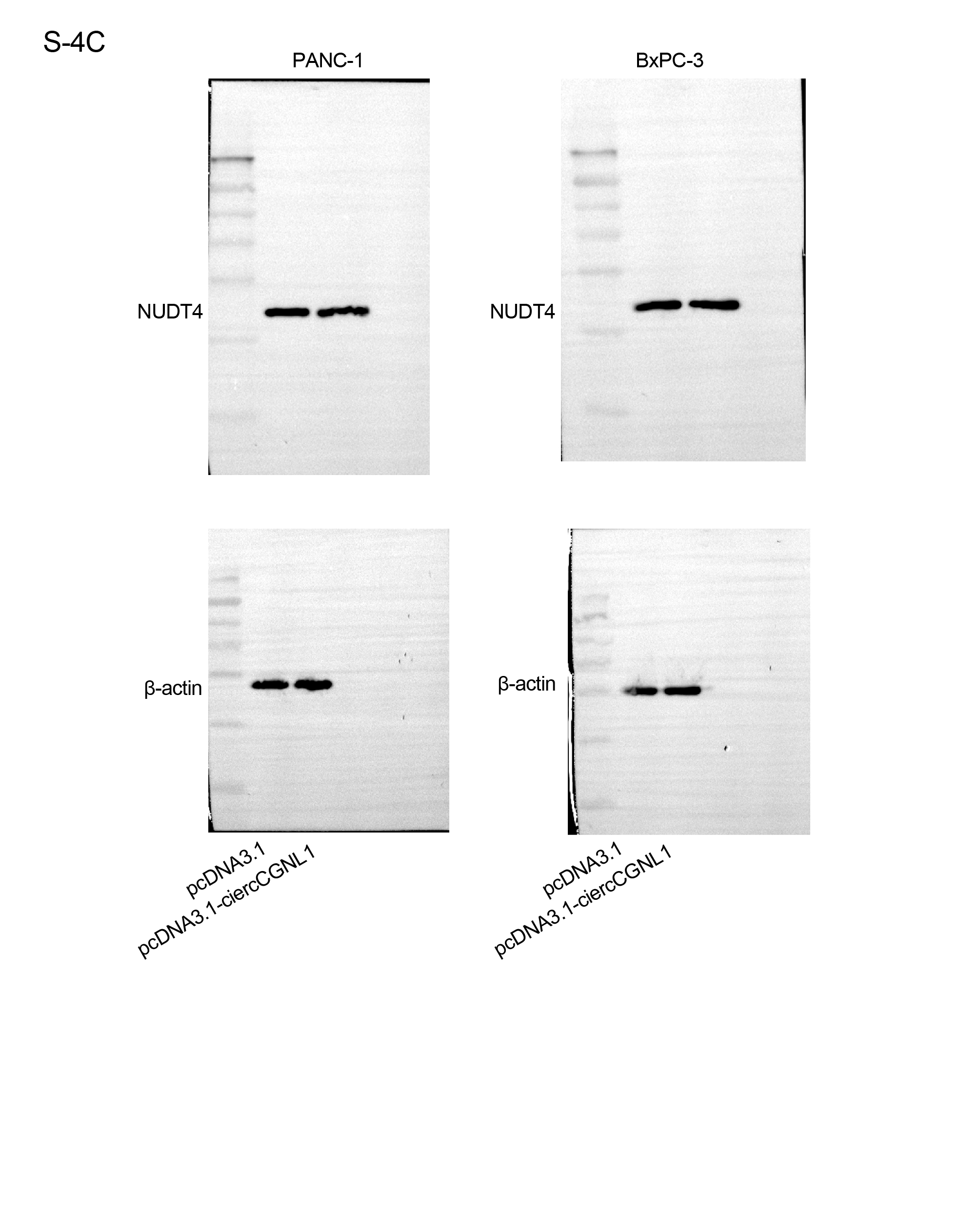

Supplement: Supplementary file 5 — Additional file 5: Supplementary Fig. 4. Relationship between NUDT4 and circCGNL1. A The Hum-mPLoc website was used to predict the subcellular location of NUDT4. B, C qRT-PCR and WB assays detected NUDT4 expression when circCGNL1 was overexpressed in PANC-1 and BxPC-3 cells. D The catRAPID website was used to predict potential binding sites between circCGNL1 and NUDT4. ns: no significance. [file 12943_2023_1923_MOESM5_ESM.jpg]

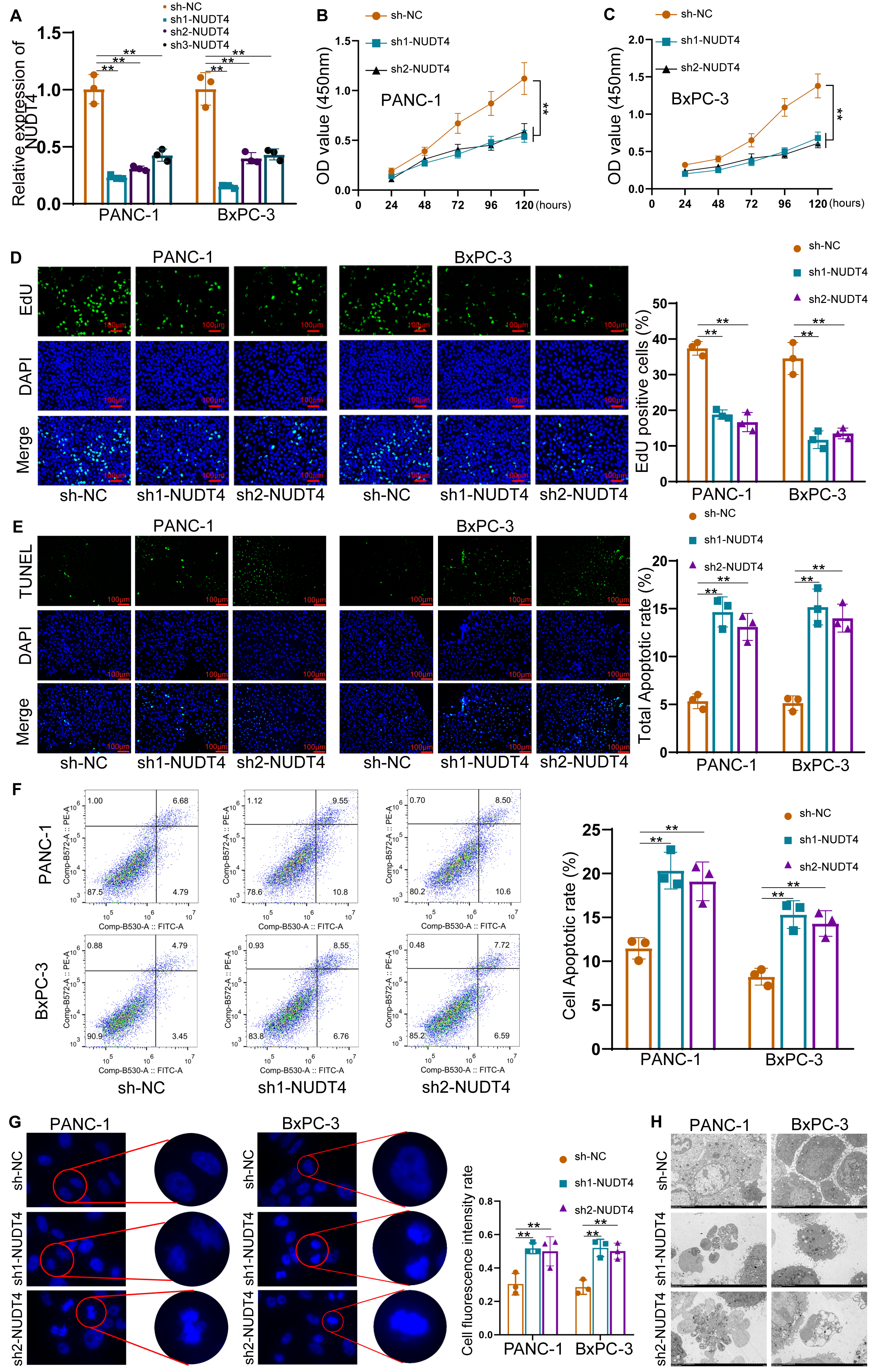

Supplement: Supplementary file 6 — Additional file 6: Supplementary Fig. 5. NUDT4 accelerated PC progression. A qRT-PCR was performed to determine the knockdown efficiency of NUDT4 shRNAs in PANC-1 and BxPC-3 cells. B-D CCK-8 and EdU proliferation assays were performed to detect cell viability when NUDT4 was inhibited in BxPC-3 and PANC-1 cells. E, F TUNEL and flow cytometric assays were performed to estimate apoptosis when NUDT4 was inhibited. G, H DAPI staining assay and TEM was used to detect PC cell apoptosis (G) and observe the cell nuclear chromatin morphological changes (H). **p < 0.01. [file 12943_2023_1923_MOESM6_ESM.tif]

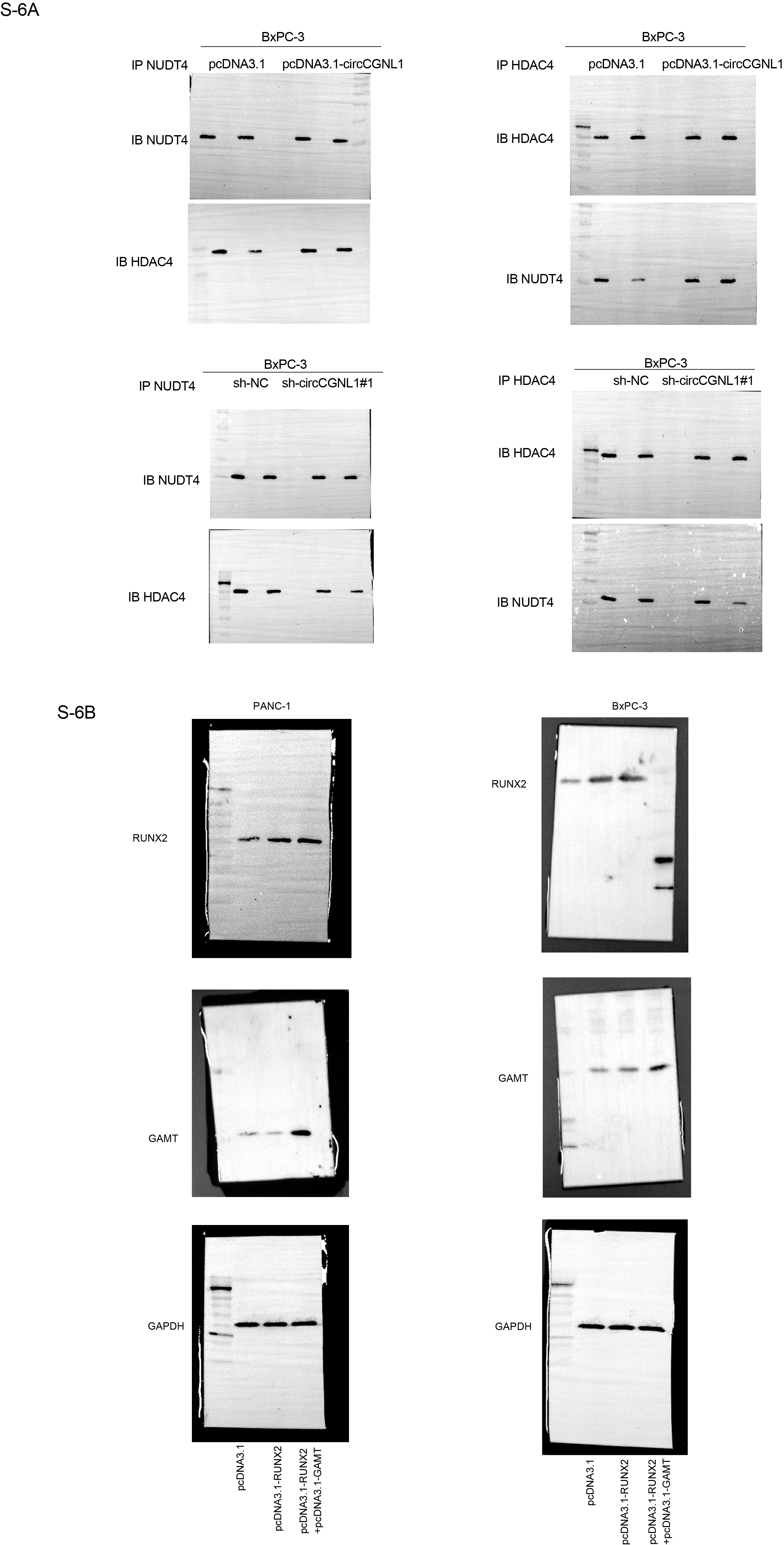

Supplement: Supplementary file 7 — Additional file 7: Supplementary Fig. 6. RUNX2 promotes PC progression depending on GAMT. A circCGNL1 was up or down-regulated in BxPC-3 cells and Co-IP assays were performed with NUDT4 or HDAC4 as bait proteins. Immunoblotting showed that the combination of NUDT4 and HDAC4 was increased by circCGNL1. B Western blot assay was used to detect RUNX2 and GAMT expression after RUNX2 transfection or RUNX2-GAMT co-transfection. C CCK-8 assays were performed to detect the viability of PANC-1 and BxPC-3 cells in different transfection groups. D, E PANC-1 and BxPC-3 apoptosis rates were detected using flow cytometry after transfecting RUNX2 or GAMT overexpression plasmid. F, G TUNEL assay was performed to examine cell apoptosis when RUNX2 or GAMT overexpressed. ns: no significance, **p < 0.01. [file 12943_2023_1923_MOESM7_ESM.jpg]

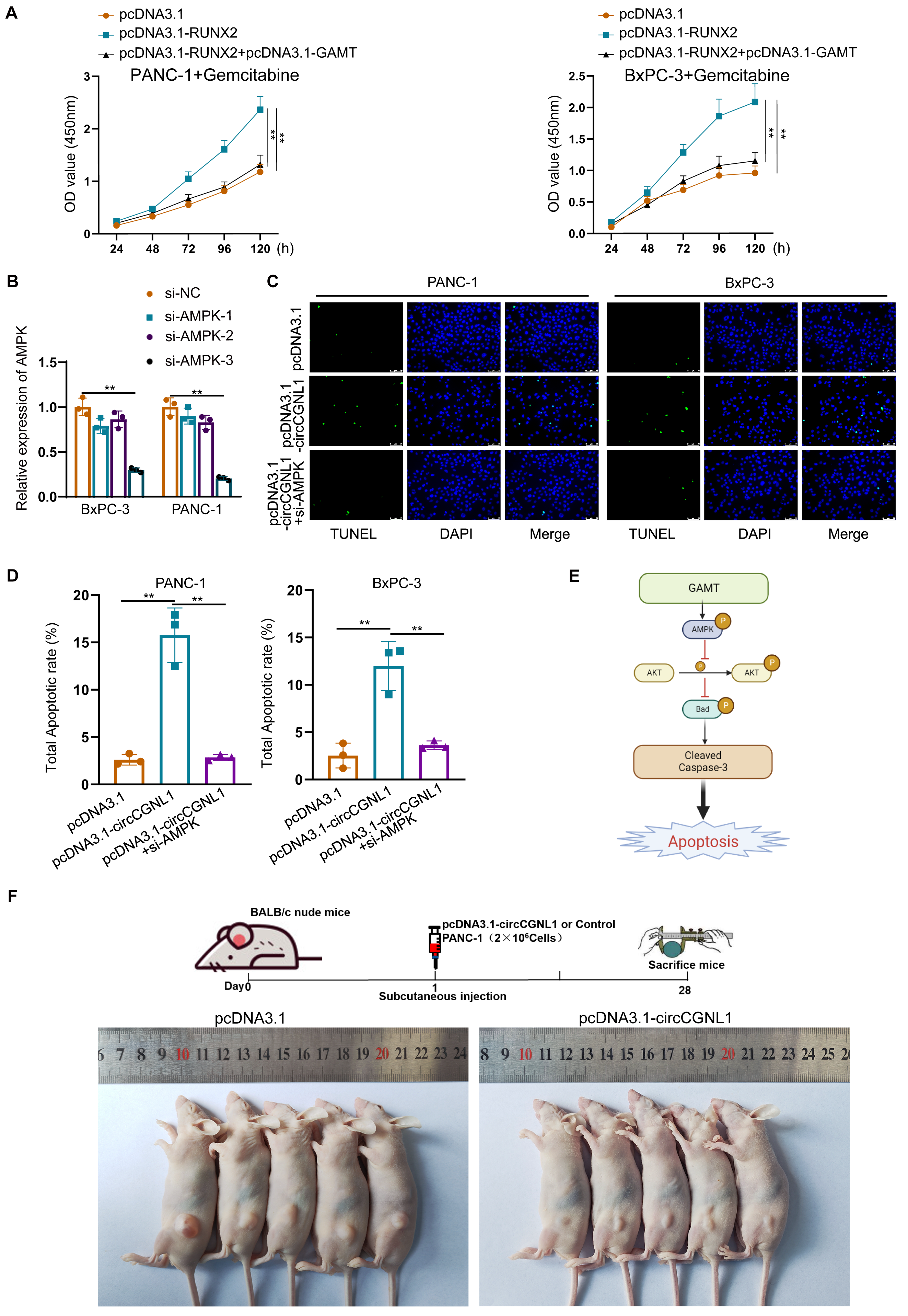

Supplement: Supplementary file 8 — Additional file 8: Supplementary Fig. 7. Subcutaneous injections in BALB/c mice. A CCK-8 assays were used to detect PC cells viability in pcDNA3.1 vector group, RUNX2 overexpressing group and RUNX2-GAMT co-overexpressing groups after gemcitabine treatment at 1 μmol/L for 24h. B AMPK siRNAs (si-AMPK-1, si-AMPK-2 and si-AMPK-3) were used to knock down AMPK in BxPC-3 and PANC-1 cells, which was identified via qRT-PCR. C, D TUNEL assay was performed to determine the PC cell apoptosis rate after overexpressing circCGNL1 or knocking down AMPK. E Schematic diagram showing the signaling pathway whereby GAMT mediates PC cell apoptosis. GAMT suppressed AKT phosphorylation by stimulating AMPK phosphorylation and subsequently suppressed Bad phosphorylation and upregulated the downstream effector, cleaved caspase-3, which induced PC cell apoptosis. In contrast, GAMT impairment diminished apoptosis. F BALB/c mice were subcutaneously injected on day 1 with 2 × 10^6^ PANC-1 cells/0.1 ml. Tumor-bearing mice from the circCGNL1-overexpression group and pcDNA3.1 control group were sacrificed at day 28 after breeding, and subcutaneous tumor tissues were harvested and measured. **p < 0.01. [file 12943_2023_1923_MOESM8_ESM.png]
